# Power Ultrasound as an Intensification Strategy in Food Biomanufacturing: Applications, Mechanisms and Perspectives

**DOI:** 10.3390/foods15193553

**Published:** 2026-10-06

**Authors:** Hui Luo, Lina Guo, Hajar Ghennami, Xinyan Zhang, Haile Ma

**Affiliations:** 1 School of Food and Biological Engineering, Jiangsu University, Zhenjiang 212013, China; 2112318149@stmail.ujs.edu.cn (H.L.); hajarghennami779@gmail.com (H.G.); 2112418177@stmail.ujs.edu.cn (X.Z.); 2 College of Life Science and Engineering, Hexi University, Zhangye 734000, China; 3 Institute of Food Physical Processing, Jiangsu University, Zhenjiang 212013, China

**Keywords:** power ultrasound, food biomanufacturing, fermentation, enzymatic hydrolysis, aging, extraction, acoustic cavitation, biocatalyst modulation, mass transfer

## Abstract

Power ultrasound has been investigated as an intensification tool for fermentation, aging, enzymatic hydrolysis, and extraction in food biomanufacturing. Treatment outcomes vary with matrix properties, application stage, acoustic exposure, and evaluation criteria, leaving optimization largely empirical. This review compares ultrasound applications across these four operations and evaluates combinations with pulsed electric fields, cold atmospheric plasma, microwave processing, and membrane separation. Potential roles in precision fermentation, microbial protein production, synthetic microbial communities, and cell-free or multi-enzyme biomanufacturing are also examined. Applications are organized by treatment stage for fermentation and hydrolysis and by product class for aging and extraction. Mechanistic evidence is interpreted according to the mechanical and chemical effects involved and the process limitation addressed. Substrate restructuring is most beneficial when physical accessibility limits conversion or compound release, whereas microbial and enzyme tolerance often constrains ultrasound intensity during fermentation and hydrolysis. Combining ultrasound with other technologies offers opportunities to exploit their complementary strengths. Emerging food biomanufacturing platforms are potential areas for expanding ultrasound applications. The value of ultrasound lies in addressing the main limitation of each process without compromising product quality. Industrial translation will require reproducible control of acoustic exposure, consistent product performance, and sufficient processing gains to justify electrical energy consumption and equipment costs.

## 1. Introduction

Food biomanufacturing uses microorganisms and enzymes to convert raw materials into foods, ingredients, and bioactive compounds. Although it encompasses a broader range of biological production and downstream operations, this review focuses on four representative processes in which ultrasound has been studied extensively. Fermentation drives biological conversion through microbial metabolism, producing foods or ingredients with modified flavor and functional properties [1]. Enzymatic hydrolysis cleaves macromolecular substrates into smaller compounds, some of which possess biological activity [2]. Aging is a downstream maturation step in which microbial proliferation largely ceases and slower chemical reactions and molecular rearrangements continue to shape and stabilize flavor [3,4]. Extraction either recovers compounds produced during bioconversion or isolates macromolecular substrates from raw materials for subsequent processing [5,6]. Across these operations, several recurring limitations provide potential targets for ultrasound. Dense raw-material structures restrict access by microorganisms, enzymes, or solvents [7], while high viscosity can slow transport within the reaction medium [8]. Low or unstable biocatalytic activity may further constrain fermentation and enzymatic hydrolysis [9]. These limitations prolong processing and reduce substrate conversion or product recovery.

Power ultrasound is used in food processing to intensify physical and biochemical transformations. As ultrasound propagates through a liquid medium, alternating compression and rarefaction drive the formation, growth, and collapse of cavitation bubbles. Bubble collapse produces mechanical effects, including microstreaming, shear forces, shock waves, and microjets, as well as localized heating and reactive species [10]. These effects can remove structural and transport barriers across different food biomanufacturing operations [9,11]. In fermentation, mild ultrasound can alter membrane permeability and microbial metabolism [12]. During aging, it promotes mass transfer and accelerates reactions involved in flavor development [13]. In enzymatic hydrolysis, ultrasound can modify substrate accessibility and enzyme conformation [14], whereas in extraction it disrupts cellular structures and facilitates solvent penetration and compound release [15,16]. However, the effects of ultrasound depend on the amount of acoustic energy delivered to the sample. Excessive exposure can intensify shear, localized heating, and radical formation, leading to enzyme inactivation, microbial cell damage, or degradation of sensitive compounds [17]. The actual acoustic energy delivered to the sample is influenced by reactor geometry, transducer configuration, treatment volume, and operating conditions [18]. Many studies report only nominal power, power density, and treatment time without quantifying the actual acoustic energy delivered to the sample. Absorbed acoustic energy is the cumulative acoustic energy absorbed by the medium during treatment, estimated by integrating calorimetrically determined acoustic power over time. Uncertainty in the absorbed acoustic energy makes it difficult to determine whether differences among studies arise from matrix properties or differences in ultrasound dose. This limits cross-study comparison and leaves process optimization and scale-up largely empirical.

Existing reviews have examined ultrasound within individual operations, including extraction [19,20], fermentation and maturation [21], alcoholic-beverage production [3], and enzyme activation [22]. These reviews provide detailed accounts of specific applications, whereas industrial food biomanufacturing often combines several operations within a production sequence. Fermentation or enzymatic hydrolysis may be followed by extraction of target compounds, while alcoholic-beverage fermentation may be followed by aging. Selecting where to introduce ultrasound therefore requires consideration of its potential contribution at different points in the production sequence, rather than its effectiveness within an isolated operation. A remaining need is to compare the available evidence across operations and treatment stages to clarify where ultrasound offers useful improvements and what limits its application. Such a comparison can support decisions on suitable intervention points and identify where further technical and economic evaluation is needed.

This review examines recent advances in power ultrasound across the established food biomanufacturing operations of fermentation, aging, enzymatic hydrolysis, and extraction. Fermentation and enzymatic hydrolysis are compared according to whether ultrasound is applied to the substrate, the biocatalyst, or the reaction process. For aging, this review summarizes the applications and mechanisms of ultrasound across different product categories, including alcoholic beverages and fermented condiments. For ultrasound-assisted extraction, applications are organized by the class of target compound. Mechanistic evidence is interpreted within each operation, preserving differences in matrix structure, biological activity, transport limitations, and the desired product changes. The review also examines combinations with pulsed electric fields, cold atmospheric plasma, microwave processing, and membrane separation. The scope is then extended to precision fermentation and microbial cell factories, biomass fermentation for microbial protein production, synthetic microbial communities, and cell-free or multi-enzyme biomanufacturing. These emerging applications are discussed with a distinction between demonstrated uses and potential opportunities. The review also examines limitations arising from incomplete mechanistic understanding, non-uniform and poorly controlled acoustic fields, and limited industrial validation. Corresponding research priorities include mechanism-based parameter selection, reproducible acoustic-field control, and pilot-scale validation supported by techno-economic analysis and life cycle assessment.

## 2. Methodology

**Figure 1:**
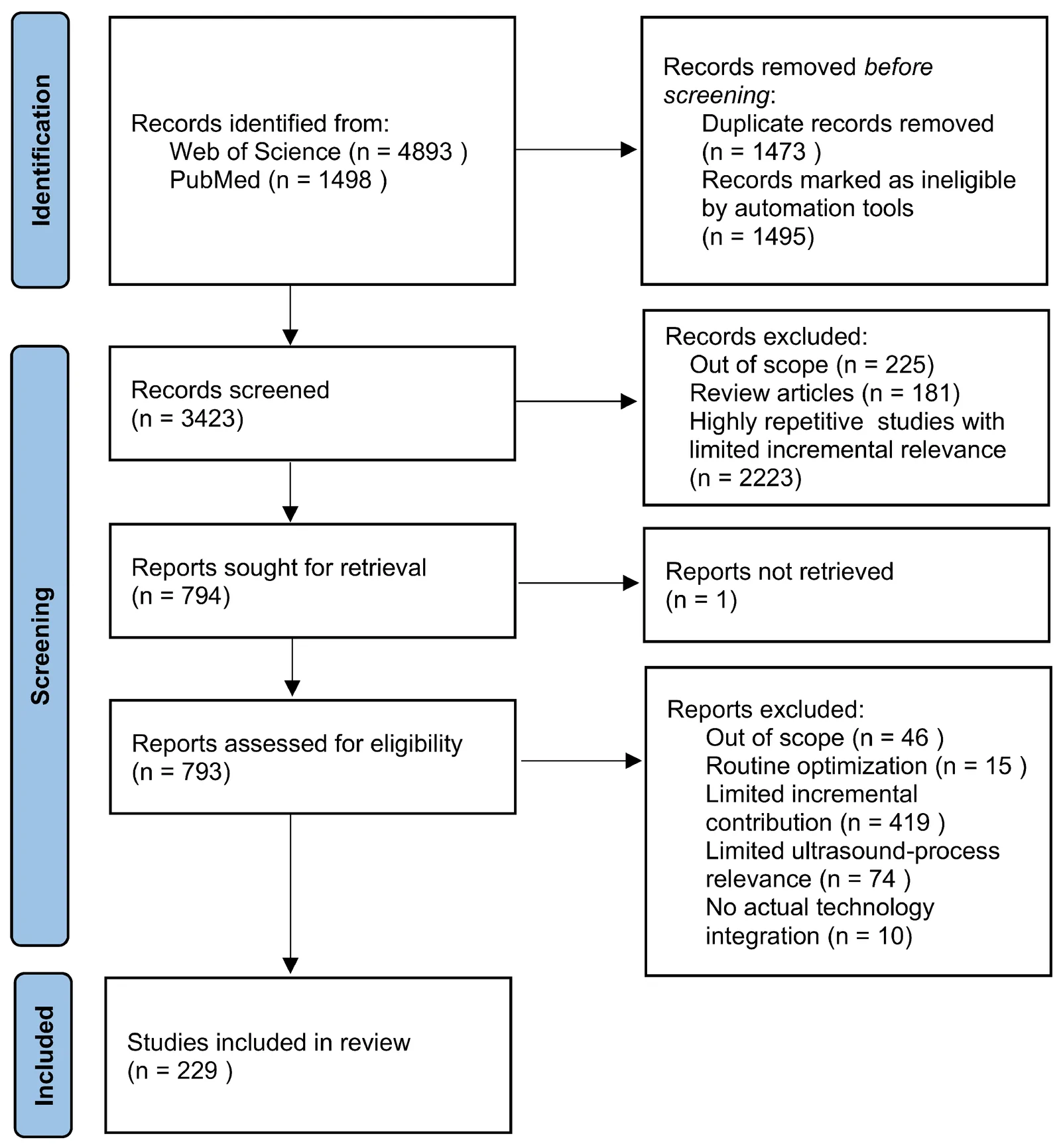
PRISMA flow diagram of the study selection process.

The literature identification and selection process is summarized in Figure 1 using a PRISMA 2020 [23] flow diagram. The Web of Science (WoS) and PubMed databases were searched for publications dated from 1 January 2015, to 7 September 2026. Core search terms included “ultrasound”, “fermentation”, “aging”, “enzymatic hydrolysis”, and “extraction” combined with food-related terms. Additional terms covered complementary technologies and emerging platforms, including “pulsed electric field”, “cold plasma”, “microwave”, “membrane separation”, “precision fermentation”, and “cell-free biosynthesis”.

The searches identified 6391 records, including 4893 from Web of Science and 1498 from PubMed. Duplicate records were removed using Zotero, and OpenAI Codex was used to flag records as ineligible before manual screening. Following title and abstract screening and full-text assessment, 229 studies were included. The selection process and exclusion reasons are presented in Figure 1. Earlier studies and background reviews already cited in the manuscript were retained separately and were not included in these counts.

## 3. Ultrasound-Assisted Fermentation

This section examines ultrasound-assisted fermentation according to the stage at which ultrasound is applied. It covers substrate pretreatment, microorganism pretreatment before inoculation, and sonication during fermentation, followed by a discussion of the underlying mechanisms. Representative studies and treatment outcomes are summarized in Table 1.

### 3.1. Applications of Ultrasound to Fermentation Substrates

Fermentation substrate pretreatment refers to the application of ultrasound to substrates before microbial inoculation. The treatment is intended to alter the physical properties of the substrate and make nutrients or structural components more accessible after inoculation. Table 1 shows varied outcomes across substrates. Millet pretreatment shortened fermentation time, whereas pretreatment of chicken liver, wheat bran, and citrus peel increased amino acid or phenolic contents after fermentation. These differences partly reflect the research objectives and endpoints selected. Because the relevant barriers differ with the physical state of the material, the available studies are discussed as liquid substrates and solid or semi-solid substrates.

Liquid substrates include dairy products, cereal slurries, and plant juices. Their continuous aqueous phase supports acoustic-energy transmission and cavitation throughout the medium [36]. Since these materials do not contain a single rigid matrix, the effects of pretreatment generally appear as changes in dispersion, rheology, or the availability of fermentable components. In cow’s milk, treatment at 150 W/L for 15 min before yogurt fermentation increased lactic, pyruvic, and acetic acids in the fermented product. Acetaldehyde, diacetyl, acetoin, and ethanol also increased [37]. The simultaneous changes in acids and aroma compounds show that pretreatment affected both acid production and the final flavor profile. White finger millet showed a different response. Pretreatment at 0.99 W/mm^2^ for 11.09 min reduced viscosity, increased water-holding capacity, and shortened acidification during lactic fermentation [26]. Lower viscosity can facilitate mixing and contact between the suspended substrate and microbial cells, making the rheological response particularly relevant in cereal slurries. In black carrot juice, sonication at 35 kHz and 60% amplitude before inoculation increased the amount of lactic acid [38]. The three studies describe different aspects of liquid-substrate pretreatment. Treatment times were relatively short, but the use of W/L, W/mm^2^, and amplitude prevents direct comparison of the acoustic energy delivered to each substrate.

In solid and semi-solid substrates, compact tissue or cell-wall architecture presents a more direct physical barrier to microbial and enzymatic access. The desired outcome depends on whether the material consists mainly of animal tissue or lignocellulosic biomass. In protein-rich animal tissues, ultrasound is applied before inoculation to modify the starting structure, after which fermentation determines how this altered matrix influences texture and precursor release. In air-dried duck meat, pretreatment reorganized muscle-fiber alignment and shifted free water toward an immobilized state, and the fermented product showed improved tenderness [39]. In chicken liver, treatment at 560 W for 30 min increased free and hydrolyzed amino acid contents by 39.38% and 41.77%, respectively, and enriched flavor-active compounds during subsequent fermentation [25].

Lignocellulosic by-products present a different problem because lignin and hemicellulose restrict microbial access to enclosed carbohydrates and phenolic compounds. Ultrasound pretreatment of wheat bran before solid-state fermentation with *Aspergillus niger* loosened the lignin–xylan network and increased total phenolic content by 21.30% on day 1 [27]. The altered structure also improved contact between the fiber and cellulase secreted during fermentation. A related response was reported for *Citrus reticulata* peel, where pretreatment increased phenolic and flavonoid accumulation and enhanced antioxidant activity after fermentation [28]. These quality indicators show that compounds retained within plant tissues became more available during fermentation.

### 3.2. Applications of Ultrasound to Microorganisms

Microorganism pretreatment refers to the application of ultrasound to fermentation microorganisms before inoculation into the substrate. The aim is to induce beneficial physiological changes that improve microbial performance during subsequent fermentation. As summarized in Table 1, ultrasound pretreatments elicit diverse physiological responses among microorganisms, including increased biomass accumulation [30], elevated cell membrane permeability [40], and enhanced activities of key enzymes [29]. These endpoints describe different aspects of the microbial response and should not be treated as equivalent measures of improvement. Biomass directly reflects growth, whereas membrane permeability and enzyme activity are physiological indicators whose practical value depends on whether they lead to faster conversion or greater product formation. The response also varies with microbial species and the growth phase at which ultrasound is applied.

Differences in cell structure and metabolism produce distinct response patterns and tolerances among strains. Among the microorganisms examined, spore-forming *Bacillus* strains have tolerated comparatively long ultrasound treatments, although the available evidence remains limited to a small number of strain-specific studies. Ultrasound treatment of *Bacillus subtilis* seed liquid for 1.5 h resulted in a 33.24% increase in bacterial biomass and enhanced extracellular protease activity [1]. A 1.5 h ultrasound treatment promoted the germination of *Bacillus licheniformis* YYC4 spores and increased bacterial biomass by 48.95% [40]. Because lactic acid bacteria do not form protective endospores, their tolerance to ultrasound can differ markedly from that of spore-forming *Bacillus* [41,42]. Reported effective treatments have accordingly been shorter than that of *Bacillus*. For *Lactobacillus plantarum*, ultrasound was applied for 30 min [30]. The treatment promoted *Lactobacillus plantarum* growth and malolactic conversion during the lag and logarithmic phases, and this metabolic response was accompanied by enhanced transformation of phenolic compounds in apple juice. The apparent difference between *Bacillus* and *L. plantarum* is consistent with their structural differences, but it cannot be attributed to spore formation alone because the studies also differed in species, physiological state, acoustic conditions, and evaluation criteria. In mixed-culture fermentation, a treatment optimized for total biomass or a single metabolite may favor one strain while suppressing another. Establishing strain-specific tolerance windows, or applying ultrasound in a staged rather than uniform manner, may be needed to intensify these fermentations without disturbing their microbial balance.

The response of microorganisms to ultrasound varies with growth phase, yet the available studies do not identify a single phase that is consistently optimal. For *Saccharomyces cerevisiae*, the logarithmic phase produced the greatest increases in Na^+^/K^+^-ATPase and total ATPase activities, by 111.62% and 67.20%, respectively [12]. Another study identified the lag phase as the most responsive, with biomass increasing by 127.03% [43]. These findings appear contradictory but arise from the use of different endpoints. Membrane transporter activity captures an immediate physiological response to ultrasound, whereas biomass reflects cumulative growth after treatment. Their maximum responses therefore need not occur during the same phase. The same distinction is evident in *Lactobacillus plantarum*. Ultrasound applied during the logarithmic phase induced cell-surface remodeling and increased H^+^-ATPase and β-glucosidase activities [29]. Treatment during the lag phase, by contrast, produced larger increases in cell growth and lactic acid production [30]. In this case, the apparent disagreement again depends partly on whether the endpoint describes cellular activation or fermentation performance.

Differences may remain even after the endpoints are considered because the studies also vary in strain, culture conditions, ultrasound parameters, and growth-phase definitions. Cross-study comparison is further weakened by the lack of a common acoustic-dose metric and by limited characterization of the physiological state of the cells at treatment. A transferable conclusion will require the same strain to be treated at defined growth phases under measured acoustic exposure and then evaluated using a common set of indicators, including viability, membrane or enzyme activity, biomass, and product formation. Until such comparisons are available, the reported optimal phase should be interpreted as specific to the endpoint and protocol used rather than as a general property of the microorganism.

### 3.3. Applications of Ultrasound During Fermentation

Ultrasound can also be applied during fermentation rather than as a pretreatment of the substrate or microorganism. When treatment is applied after inoculation, it acts on an evolving system in which substrates, microbial cells, and metabolites coexist. Its effects may therefore extend beyond modification of the starting material to changes in microbial activity and transport during fermentation [44]. The in-process studies in Table 1 report improvements in both fermentation performance and product properties. Yogurt treatment shortened fermentation time and improved water retention, whereas dough treatment was associated with changes in protein interactions and network stability. These findings indicate that the value of in-process ultrasound extends beyond faster microbial conversion to modification of the fermented matrix. The response depends on the physical state of the medium. Acoustic waves can propagate through both liquid and solid materials, whereas sustained cavitation is favored by a sufficiently continuous liquid phase. Matrix density, free-water content, viscosity, and the presence of gas–liquid or solid–liquid interfaces influence acoustic attenuation, bubble behavior, and mechanical-energy transfer [45,46]. Submerged fermentation (SmF) proceeds in a free-flowing liquid medium, where cells are suspended in a continuous aqueous phase. Solid-state fermentation (SSF) proceeds on a moist solid substrate with little free water and no continuous liquid phase. This limitation is reflected in SSF studies that sonicate the inoculum before it enters the solid matrix rather than apply ultrasound directly during fermentation [47]. Semi-solid matrices lie between these conditions. Their limited free water weakens cavitation, but deformable networks and multiphase interfaces can remain responsive to vibration and shear. The distinction among SmF, SSF, and semi-solid fermentation therefore reflects differences in how acoustic energy reaches the fermenting components, rather than water content alone.

In SmF, the continuous liquid phase transmits acoustic energy efficiently, so in-process ultrasound acts directly on the suspended cells. Under appropriate conditions, this in-process application can intensify liquid fermentation more effectively than pretreating the substrate alone. In millet lactic acid fermentation, ultrasound pretreatment of the substrate shortened fermentation from 9 h to 5.3 h, whereas ultrasound applied after inoculation shortened it to 2.5 h, giving the highest rate constant for lactic acid production [26]. Ultrasound applied during yogurt fermentation likewise reduced the fermentation time and produced a smoother texture by modifying the protein network [33]. However, improved fermentation efficiency does not necessarily lead to better texture. In probiotic yogurts, post-inoculation ultrasound shortened fermentation time but did not improve hardness or water-holding capacity relative to the untreated control, whereas pre-inoculation treatment improved both properties [48]. The mode of ultrasound application also affects yogurt fermentation performance. In one study, ultrasound-bath treatment did not accelerate fermentation, whereas direct probe treatment shortened fermentation time. This difference may reflect acoustic energy losses through the bath medium and vessel wall, whereas an immersed probe delivers energy directly into the fermentation medium [41].

Scaling up ultrasound intensification of liquid fermentation remains a challenge. Most SmF studies treat only small volumes, such as sea buckthorn juice in a 6 L ultrasonic bath [49] or 50 mL of inoculated buffalo milk under a probe [50]. Such volumes cannot predict performance at production scale. In a 7.5 L fermenter, ultrasound increased the activities of key glycolytic enzymes (hexokinase, phosphofructokinase, and pyruvate kinase) and raised ethanol yield by about 30% [44]. This experiment moved beyond small laboratory samples, but the working volume remained well below that of industrial production. Increasing vessel size also increases the distance between the transducer and much of the medium, which can create strongly treated regions near the acoustic source and weakly treated regions elsewhere [51]. Scale-up studies should evaluate distributed transducers or continuous-flow configurations and report acoustic-field distribution, absorbed energy, energy consumption per unit of product, and operating cost. Without these measurements, a gain obtained in a small reactor cannot be separated from the particular geometry and energy distribution of that device.

In SSF, the scarcity of free water and the absence of a continuous liquid phase suppress sustained cavitation, so ultrasound is seldom applied in-process and is used mainly as a pretreatment before SSF. In practice, the seed culture may be sonicated before inoculation [1], the substrate may be sonicated before fermentation [27], or both may be treated separately beforehand [28]. Acoustic waves can enter a moist solid matrix, but its heterogeneous structure attenuates and scatters the energy and does not provide the liquid environment required for repeated bubble growth and collapse [52]. The available SSF studies consequently provide evidence for pre-fermentation treatment of the substrate or inoculum, rather than for ultrasound intensification of the ongoing solid-state process.

Semi-solid systems behave differently. In dough fermentation, gases and metabolites released by yeast continually alter pH and interfacial tension, keeping the gluten network in dynamic rearrangement [35]. Under such conditions, ultrasound-induced protein unfolding and additional gluten cross-linking were associated with greater network stability [53]. This response can occur despite the limited amount of free water because the deformable gluten network and the gas cells generated during fermentation provide multiphase interfaces that respond to vibration and local shear. The behavior of dough shows that free-water content alone does not determine whether an in-process treatment will be effective; matrix deformability and interfacial structure also influence how acoustic energy is transferred.

### 3.4. Mechanisms of Ultrasound-Assisted Fermentation

**Figure 2:**
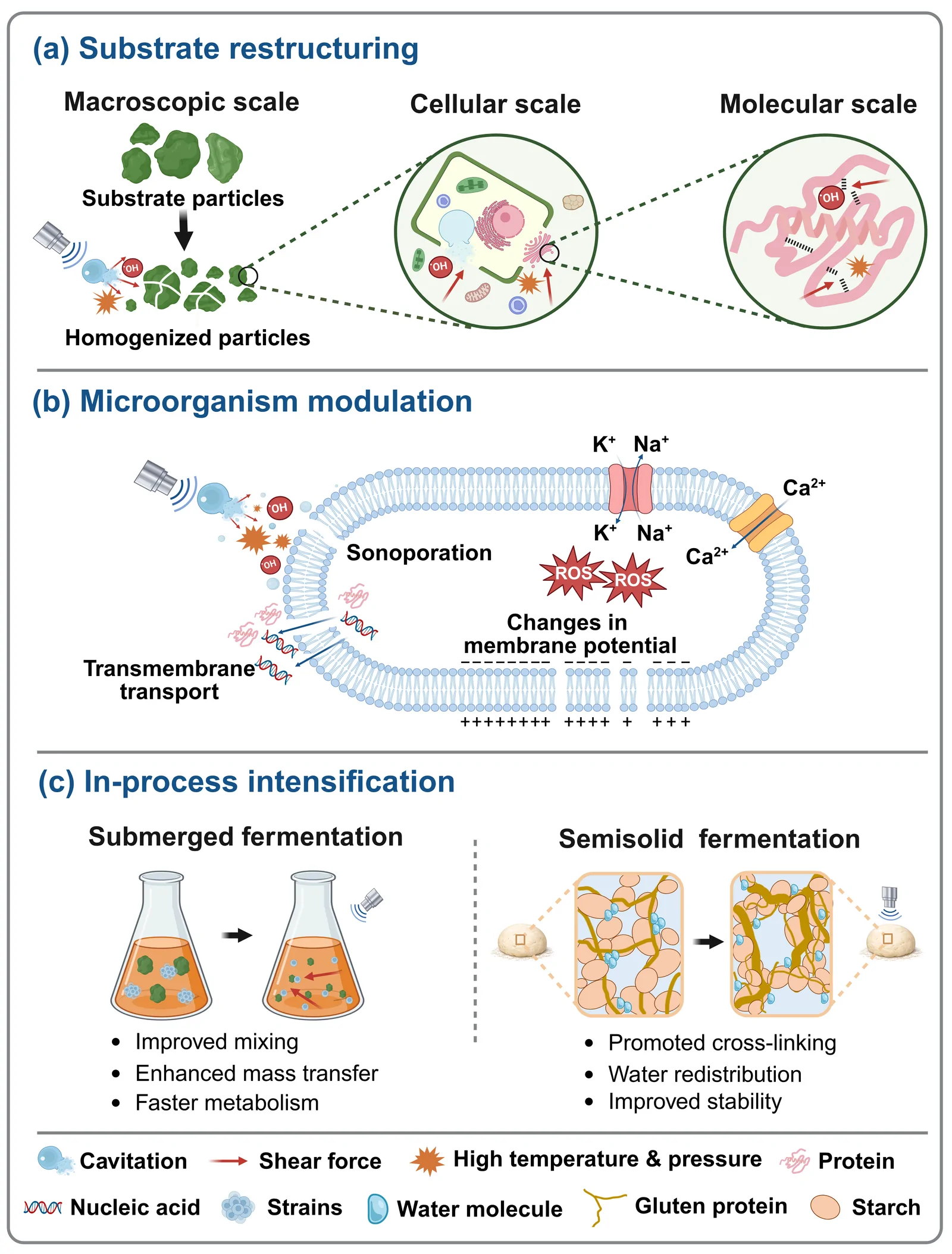
Mechanisms underlying ultrasound-assisted fermentation. (**a**) Ultrasound restructures substrates through particle homogenization, cell-wall disruption, and macromolecular unfolding, improving accessibility. (**b**) Cavitation-induced sonoporation and oxidative stress alter membrane permeability, ion transport, membrane potential, and microbial metabolism; excessive exposure causes intracellular leakage. (**c**) In submerged fermentation, ultrasound enhances mixing and mass transfer. Cavitation is limited in solid-state fermentation, whereas semi-solid matrices such as dough respond mainly to vibration and shear, promoting cross-linking, water redistribution, and network stability.

Ultrasound affects fermentation mainly through acoustic cavitation. Bubble oscillation and collapse generate mechanical effects, including shear, microstreaming, shock waves, and microjets. Cavitation also produces reactive radicals through sonochemical reactions, together with transient local increases in temperature and pressure. These effects act on the substrate, the fermentation microorganisms, and the reaction system. At the substrate, ultrasound changes physical structure and molecular accessibility. Its action on microorganisms alters membrane properties and physiological activity, while changes in the reaction system affect transport and conversion during fermentation (Figure 2). The contribution of each route depends on the substrate, microbial tolerance, physical state of the medium, and acoustic exposure.

Ultrasound pretreatment reshapes the structure of fermentation substrates on macroscopic, cellular, and molecular scales, thereby influencing substrate fermentability and the quality of the final product. At the macroscopic scale, the shock waves and microjets generated during bubble collapse impose intense local stresses that fracture and erode particles, while microstreaming disperses aggregates [54]. As a result, particle size decreases, and the substrate becomes more homogeneous, increasing the effective contact area between microbial cells and the substrate [26]. At the cellular scale, cavitation-induced microjets and shear forces can disrupt cell walls when local stresses are sufficient. In lignocellulosic materials, repeated compression and rarefaction further weaken the lignin-hemicellulose network, improving the accessibility of cellulose and hemicellulose to microbially secreted enzymes [27]. At the molecular scale, hydrodynamic stresses and transient heating may disturb the noncovalent interactions that stabilize protein structure. Cavitation-derived radicals may also contribute through oxidative modification. Partial protein unfolding may expose buried hydrophobic groups and cleavage sites, potentially influencing microbial proteolysis, peptide release, and flavor formation during fermentation [25]. Despite operating at different structural levels, these responses follow a common principle. Structural modification occurs when the local cavitation force exceeds the characteristic mechanical strength of the target. The dominant response is governed by substrate architecture. Brittle or lignocellulosic materials respond mainly through fragmentation and cell-wall disruption, whereas protein-rich substrates respond through conformational rearrangement.

Ultrasound alters the fluidity and permeability of the microbial cell membrane, which in turn reshapes cellular metabolic activity. The membrane is modified through both the mechanical and chemical effects of acoustic cavitation. Mechanically, shear forces and microstreaming from periodic oscillation cause reversible membrane deformation and a transient rise in permeability, a process termed sonoporation [1]. In *Bacillus licheniformis*, scanning electron microscopy confirmed sonoporation was accompanied by increased culture conductivity, leakage of proteins and nucleic acids, elevated intracellular Ca^2+^, and altered membrane potential [40]. Chemically, cavitation-derived radicals can promote membrane-lipid peroxidation and disturb membrane integrity, thereby affecting membrane permeability and cellular activity [55]. Moderate ultrasound thus promotes transmembrane transport and ion exchange, whereas excessive exposure causes irreversible membrane damage. These permeability changes have been associated with downstream metabolic changes. Changes in membrane potential may influence the proton motive force and ATP-generating capacity, and have been accompanied by altered intracellular Ca^2+^ levels [12,40]. Enhanced Na^+^/K^+^-ATPase activity may facilitate Na^+^ and K^+^ transport and help maintain osmotic balance. Intensified intracellular-extracellular exchange is a possible contributor to enhanced energy metabolism and extracellular enzyme activity, such as protease activity [40]. Accelerated metabolism may also raise reactive oxygen species levels, which influence cellular signaling at moderate levels but cause oxidative stress and irreversible damage when excessive [56]. Membrane permeabilization offers a link between ultrasound exposure and downstream changes in energy metabolism, enzyme secretion, oxidative signaling, and ultimately fermentation performance. Its benefit is confined to a moderate intensity window. The same permeabilization, ion flux, and radical signaling that stimulate metabolism become destructive when ultrasound is too intense. It makes microbial cells the most dose-sensitive target of ultrasound in fermentation.

The way ultrasound intensifies the fermentation process depends on the physical state of the system. In SmF, the continuous liquid phase sustains cavitation, whose microstreaming, microjets, and turbulence improve mixing and accelerate the diffusion of nutrients, gases, and metabolites [26]. This enhanced mass transfer increases substrate-cell contact. Together with the membrane and metabolic changes above, it raises the apparent rate constant and speeds substrate conversion and product accumulation [12]. This benefit is bounded by a dose window. Excessive cavitation generates shock waves and high shear that disrupt membranes, release intracellular contents, and inactivate cells, eroding the kinetic advantage [40]. SSF presents a different acoustic environment. Sound waves can propagate through moist solids, but heterogeneous particles scatter and attenuate the energy, while the limited continuous liquid phase restricts repeated bubble growth and collapse [52]. Classical cavitation is consequently weaker and less uniformly distributed than in SmF, which limits the use of ultrasound during the ongoing process. Semi-solid, multiphase systems such as fermenting dough remain responsive because acoustic energy can couple with deformable protein networks and gas–liquid interfaces. Under these conditions, mechanical vibration, microscale shear, and interfacial disturbance may contribute more than sustained cavitation. Ultrasound treatment has been associated with sulfhydryl-to-disulfide cross-linking, changes in protein secondary structure, and redistribution of water toward more tightly bound states. These modifications have been associated with a stronger gluten network and improved capacity to retain fermentation gases [35,53]. The same studies report changes in yeast activity and CO_2_ production, suggesting that ultrasound may influence both gas retention by the matrix and gas generation by the microorganism. Direct evidence that thinning of the boundary layer around yeast drives transmembrane transport remains limited, so this pathway should be treated as a mechanistic explanation rather than an established sequence.

## 4. Ultrasound-Assisted Aging

Aging allows many fermented foods to develop their characteristic color, aroma, taste, and mouthfeel through gradual oxidation, esterification, polymerization, and Maillard-related reactions [57,58]. These transformations may require months or years of controlled storage, increasing warehouse demand, inventory time, and production cost. Ultrasound has been investigated as a means of shortening the time needed to achieve selected aging-related changes by enhancing mass transfer and changing the rates of aging-related reactions. The intended outcome also differs between product groups. In alcoholic beverages, ultrasound aging is used mainly to stabilize color, reshape the volatile profile, and reduce astringency. In fermented condiments, the treatment is directed toward faster development of aging-related changes in color, aroma, and taste. Because the two groups differ in composition and aging objectives, their applications and mechanisms are discussed separately in this section.

### 4.1. Applications of Ultrasound to Alcoholic Beverage Aging

Wine is extensively studied alcoholic beverage in ultrasound-assisted aging. Available studies report that sonication can accelerate selected changes associated with natural aging [59]. Such comparisons are usually based on specific color, volatile, phenolic, or sensory indicators. Reported color effects include improved chromatic stability and clarity and reduced undesirable browning [60]. Ultrasound treatment can nevertheless cause pigment losses in wine. In a study of ultrasound-assisted aging on lees, total pigment concentrations in sonicated wines were 47.8–55.9% lower than in the corresponding non-sonicated controls. The reduction mainly involved monomeric anthocyanins [61]. Ultrasound treatment has also increased fruity esters and other aging-related volatiles while reducing compounds associated with sharp or immature notes [62]. Changes in volatile concentration describe aroma composition, but a more balanced bouquet can only be established when chemical analysis is supported by sensory evaluation. In terms of mouthfeel, ultrasound can alter the structure and composition of wine phenolics, affecting their binding to salivary proteins. The response of wine phenolics depends on absorbed energy density. Phenolic–protein binding decreased at low energy densities (0–33.07 J/mL) but increased at higher levels (66.14–165.34 J/mL) [63]. Moderate ultrasound exposure may therefore improve mouthfeel, whereas excessive energy input could increase astringency and compromise sensory quality.

Ultrasound has also been tested in mead and grain-based fermented beverages. During the aging of astragalus mead, ultrasound produced a larger increase in volatile esters than the other non-thermal treatments compared in the same study. Ester content rose by 191.30%, accompanied by stronger floral and fruity aroma attributes [64]. In Chinese rice wine, ultrasound increased volatile esters, total esters, and total acids [13]. The ester response is consistent with aroma maturation, whereas the rise in total acids requires separate sensory evaluation because a higher acid content may either improve flavor balance or produce excessive sourness. Ester accumulation has been observed across grape wine, mead, and rice wine. The evidence identifies ester evolution as a recurring response.

The studies considered to date are concentrated in beverages containing less than approximately 15% alcohol by volume. Evidence for spirits and other high-alcohol systems above 35% v/v remains scarce, and an ethanol range suitable for ultrasound-assisted aging has not been established. Results obtained from wine, mead, and rice wine cannot be transferred directly to distilled spirits because ethanol concentration, initial congener composition, and the intended aging profile all differ. Validation in high-alcohol products should compare several ethanol concentrations under the same measured acoustic exposure and track desirable aroma development together with oxidation products and volatile losses. A broader limitation concerns the criteria used to claim accelerated aging. Full sensory assessment, storage stability, and comparison with naturally or barrel-aged controls are still needed, especially because ultrasound does not reproduce wood-component extraction and slow oxygen exposure merely by matching a selected set of chemical markers. Evidence of industrial usefulness will also require treatment-volume and energy-consumption data rather than aging-time reduction alone.

### 4.2. Applications of Ultrasound to Fermented Condiment Maturation

The quality of fermented condiments such as soy sauce and vinegar continues to develop after the main fermentation stage as aroma, taste, and color compounds undergo further transformation. Production of high-salt, liquid-state soy sauce generally requires 6 to 12 months, and aroma often develops more slowly than taste [65]. Ultrasound has been examined at both early and late stages of this extended process. During active moromi fermentation, low-intensity ultrasound (60 W/L) enhanced caramel-like, fruity, alcoholic, and floral notes and shortened the reported fermentation period by about 90 days [66]. Although this treatment preceded aging, it may have altered the pool of flavor precursors available for subsequent maturation. A different approach applied high-intensity ultrasound (39.48 W/cm^2^) during aging at 90 °C. This treatment increased the concentrations of esters, pyrazines, and ketones [67]. These two strategies show that ultrasound can serve either as a gentle trigger during active fermentation or as an intensive finishing treatment during aging.

Ultrasound has shown similar promise for vinegar. In Zhenjiang vinegar, 75 min of treatment produced changes in selected aging indicators reported to resemble those associated with 2 to 3 years of natural aging [68]. Coupling ultrasound with micro-oxygenation gave further gains, lowering perceived acidity while raising esters, heterocyclic compounds, and aromatic aldehydes [69]. Ultrasound has also increased the antioxidant activity of vinegar by enriching polyphenols and flavonoids [70]. These results are encouraging, but the evidence base has clear limits. Similarity in selected chemical or sensory indicators does not establish equivalence in overall quality or subsequent storage behavior. Whether ultrasonically aged products truly match traditionally aged ones remains unconfirmed. Coverage is also narrow, since work has focused almost entirely on soy sauce and vinegar. Condiments such as fish sauce, miso, and doubanjiang have scarcely been examined, even though their high-salt, high-protein, or solid-state matrices may respond quite differently. Extending ultrasonic aging to these products, and validating it against full quality and safety criteria rather than flavor markers alone, are the main gaps that remain.

### 4.3. Mechanisms of Ultrasound-Assisted Aging

During post-fermentation aging, microbial growth has generally subsided, and ultrasound acts mainly on the reaction system. Cavitation-induced microstreaming improves mixing and shortens transport distances where reactant diffusion is limiting. Radical formation and transient local heating can also change reaction rates and product distributions. The relative contribution of these effects depends on the composition and physical properties of the product.

**Figure 3:**
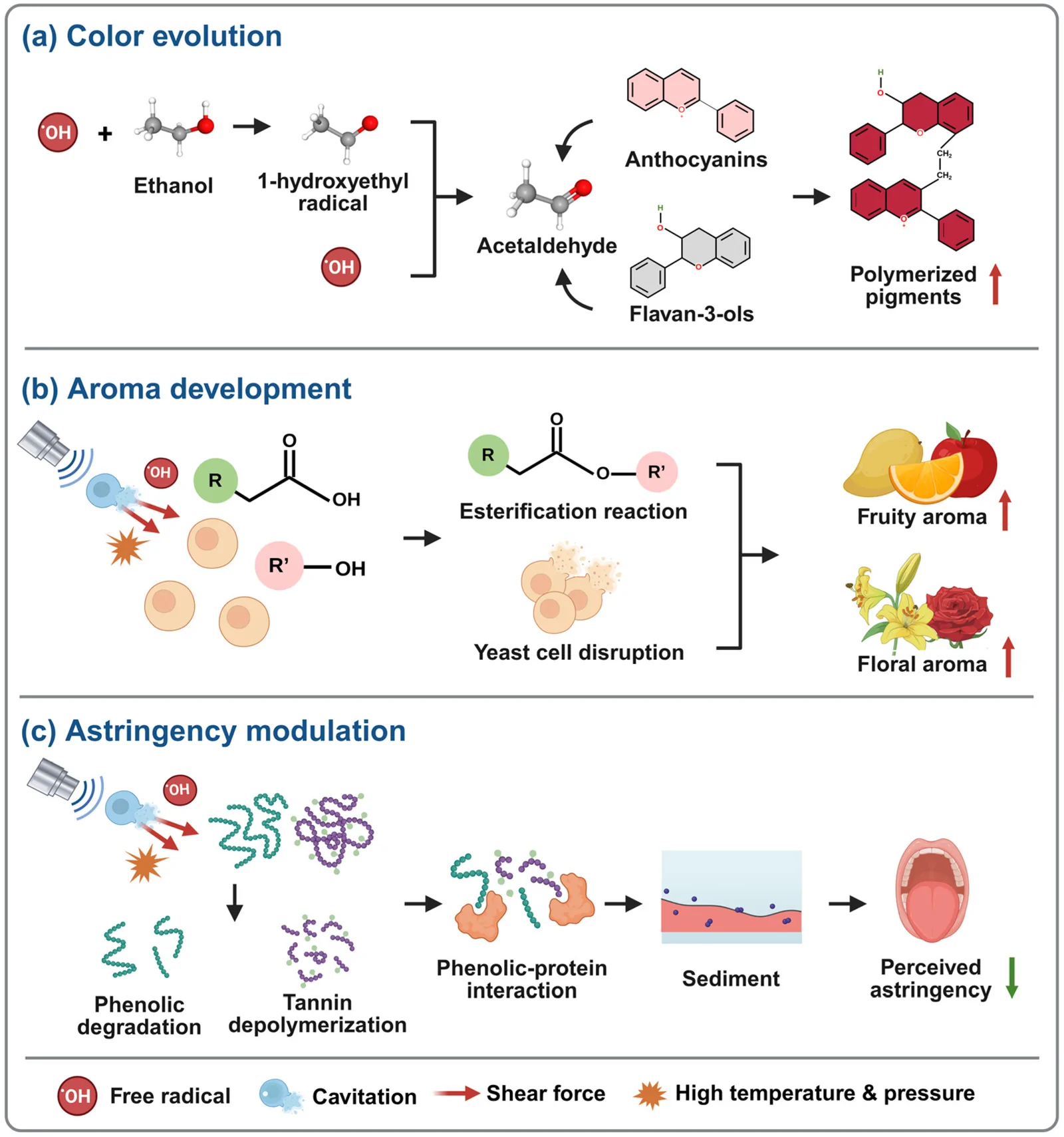
Mechanisms of ultrasound-enhanced wine aging. Cavitation and its associated free radicals, high temperature and pressure, and shear forces accelerate aging via three pathways. (**a**) Hydroxyl radicals and ethanol-derived 1-hydroxyethyl radicals promote acetaldehyde formation, which bridges anthocyanins and flavan-3-ols into polymerized, color-stabilizing pigments. (**b**) Cavitation accelerates esterification (increasing fruity esters) and disrupts yeast cells (releasing precursors of floral alcohols). (**c**) Hydroxyl radicals degrade phenolics and depolymerize tannins, weakening phenolic-protein interactions and reducing sediment, thereby lowering perceived astringency. Black arrows indicate the sequence of reactions or processes within each panel. Red arrows indicate an increase in the corresponding substances or products, and the green arrow indicates a decrease in perceived astringency.

In alcoholic beverages, ultrasound affects the interconnected oxidation, polymerization, esterification, and phenolic reactions that shape color, aroma, and mouthfeel (Figure 3). Color evolution in grape wine provides a clear example of radical-mediated chemistry. Hydroxyl radicals generated during cavitation react with ethanol to form 1-hydroxyethyl radicals [71]. Subsequent reactions involving these radicals contribute to acetaldehyde formation. Acetaldehyde can bridge anthocyanins and flavan-3-ols, producing polymeric pigments that are more resistant to bleaching and other forms of color loss [72]. Ultrasound has been reported to favor polymeric pigment formation and improve color stability [60]. Aroma development responds to both enhanced mixing and changes in reaction conditions. Cavitation can bring alcohols and organic acids into closer contact, while transient local heating can favor esterification. These effects offer a possible explanation for increased levels of compounds such as ethyl acetate, ethyl hexanoate, and ethyl octanoate. Ultrasound can also affect the transformation of aroma precursors and other volatile compounds, producing profiles with stronger floral and fruity notes and fewer harsh alcohol-related notes [62]. Increased ester concentrations have been reported in grape wine, rice wine, and mead, making ester accumulation a recurring response among the beverages studied. Total ester content alone cannot establish aroma improvement because individual esters differ in odor threshold and sensory contribution. Volatile losses and excessive formation of particular esters may also offset the benefits of faster esterification. Phenolic transformation overlaps with color development but is especially relevant to astringency. Cavitation-derived radicals can modify tannins and other phenolic compounds, changing their molecular weight distribution and affinity for salivary proteins. Because astringency depends more directly on phenolic–protein binding than on total phenolic concentration, changes in phenolic structure may improve mouthfeel even when the overall phenolic content changes little. Ultrasound-induced phenolic transformations have been associated with weaker phenolic–protein interactions and a smoother sensory profile [63].

Fermented condiments provide a more heterogeneous reaction environment. In soy sauce, high concentrations of amino acids, reducing sugars, and salts can restrict molecular transport and create local concentration gradients. Microstreaming and turbulence improve contact among reactants, allowing aging-related reactions to proceed more evenly through the liquid matrix. Sonochemical reactions occur at the same time. Cavitation-derived radicals and transient local heating may promote Strecker degradation and the formation or rearrangement of reactive carbonyl intermediates involved in the Maillard reaction [21]. Vinegar differs from soy sauce in composition and in the reactions that dominate maturation. Its aging involves esterification and oxidation, together with the formation of Maillard-derived heterocyclic compounds in traditional cereal vinegars. During ultrasonic aging of Zhenjiang vinegar, reducing sugars and amino acids declined in patterns resembling those observed during natural aging. Pyrazines, furfural, trimethyloxazole, and other heterocyclic compounds accumulated more rapidly and contributed to roasted and savory aroma notes [68]. This resemblance supports the acceleration of aging-related chemistry, but it does not prove that ultrasound reproduces the complete sequence of natural aging reactions. Experiments using simplified Maillard systems provide additional mechanistic support. In a fructose–glycine model, ultrasound promoted the formation of reaction intermediates and late-stage browning products, with cavitation-derived reactive species proposed to contribute to these changes [73]. Such models isolate individual reactions but do not reproduce the acidity, salts, oxygen availability, and diverse precursors present in fermented condiments.

Most proposed aging mechanisms are inferred from changes in product composition rather than measured directly. Few studies quantify radical generation, local thermal effects, dissolved oxygen, or transport kinetics under the same treatment conditions. Radical-scavenging experiments and matched thermal controls are needed to distinguish sonochemical effects from heating.

## 5. Ultrasound-Assisted Enzymatic Hydrolysis

This section examines the applications and mechanisms of ultrasound-assisted enzymatic hydrolysis from three perspectives, organized according to the stage at which ultrasound acts: ultrasound pretreatment of the hydrolysis substrate, ultrasound activation of enzymes, and ultrasound during enzymatic hydrolysis. Representative findings are summarized in Table 2.

### 5.1. Applications of Ultrasound to Hydrolysis Substrates

Ultrasound pretreatment is used to modify substrate structure with the aim of improving enzyme accessibility and subsequent hydrolysis. Because the principal components and the structural barriers that limit enzymatic attack differ among substrates, the purpose of ultrasound pretreatment is substrate-dependent. The substrate-pretreatment studies in Table 2 reflect these different objectives. The studies reviewed here are discussed according to two major substrate groups, carbohydrate-rich and protein-rich materials.

Among carbohydrate-rich materials, current studies have focused mainly on lignocellulosic food and agricultural residues. Ultrasound pretreatment is used to weaken their recalcitrant structure and improve enzymatic saccharification. Thermal extrusion combined with ultrasound (40 kHz, 500 W, 1.5 h) was used to pretreat rice husk [87]. This combined physical pretreatment, which did not require chemical reagents, raised the reducing-sugar yield after enzymatic hydrolysis by 35%. The improvement was associated with partial lignin removal, lower cellulose crystallinity, and greater substrate porosity and surface area, which increased cellulose accessibility to enzymes. Another combined treatment coupled ultrasound with deep eutectic solvent to pretreat corncob biomass [88]. The ultrasonic step enhanced lignin removal and the subsequent enzymatic hydrolysis of the delignified fiber. For brewer’s spent grain, ultrasound pretreatment increased the yield of fermentable sugars after enzymatic hydrolysis, supporting the use of this residue as a fermentation feedstock [74]. Notably, in most of these studies ultrasound was combined with another pretreatment, such as extrusion or a deep eutectic solvent. This pattern suggests that ultrasound may be more effective as a complementary step than as a standalone treatment for recalcitrant biomass, although direct comparisons remain limited.

For protein-rich substrates, current studies have focused mainly on underutilized proteins and protein-containing by-products. Oyster protein powder was pretreated before enzymatic hydrolysis using a 20 kHz ultrasonic cell disruptor [2]. Ultrasound facilitated the formation of low-molecular-weight peptides and enhanced their antioxidant activity. Ultrasound pretreatment has also been examined as a means of improving the utilization of edible bird’s nest fragments [89]. The protein material was treated for 30 min at 90% amplitude before hydrolysis. Ultrasound pretreatment reduced the viscosity of the resulting hydrolysates. Their lipoxygenase- and α-amylase-inhibitory activities increased by more than 100% and 43%, respectively. Peptides were also produced from surplus mulberry residues after ultrasound pretreatment, and the resulting hydrolysates inhibited HepG2 cell growth and induced cell death in vitro [90]. The studies therefore emphasize changes in peptide activity rather than a simple increase in peptide yield. Reported improvements include antioxidant and enzyme-inhibitory activities, as well as antiproliferative effects in a cell model. The type of activity cannot be generalized across substrates because the protein source and protease determine the peptide sequences released during hydrolysis. For example, ultrasound pretreatment improved the ACE-inhibitory activity of whey protein hydrolysates produced with bromelain but not those produced with papain [91]. Structural modification alone therefore does not guarantee greater release of peptides with the desired activity.

### 5.2. Applications of Ultrasound to Enzyme Activation

Under appropriate conditions, ultrasound can directly enhance the catalytic activity of enzymes, a phenomenon known as ultrasound activation. In this mode of application, the free enzyme is treated before hydrolysis, and the resulting change in activity is measured after sonication. The response varies considerably among studies. This variation is associated with both enzyme properties and ultrasound conditions, which are considered below.

Enzyme structure appears to contribute to differences in ultrasound responsiveness. Reported activity increases have generally been larger for carbohydrases and lipases than for proteases, although these values were obtained under different treatment and assay conditions. Ultrasound treatment at 20 kHz and 38 W/L increased cyclodextrin glycosyltransferase activity by 93% and was accompanied by conformational changes in the enzyme [78]. Lipase activity increased to 1.6 times the untreated level after ultrasound treatment at 22 kHz and 11.38 W/cm^2^ for 25 min [80]. Reported increases in protease activity have generally been smaller. Early studies indicated that ultrasound treatment increased alkaline protease activity by only 5.8% [92]. Although more recent studies have shown that ultrasound can enhance the activities of proteases such as Flavourzyme, Neutrase, and Alcalase by up to approximately 20% [14], the extent of activation remains lower than the increases reported for carbohydrases and lipases. These percentages do not yet establish an inherent difference among enzyme classes because the studies used different acoustic conditions, enzyme concentrations, and activity assays. Ultrasound-induced activation has been attributed to changes in enzyme dispersion, dissociation, and conformation [22]. Table 2 shows that activation can accompany opposite changes in aggregation. The protease study reported increased particle size and polydispersity, whereas both decreased for cyclodextrin glycosyltransferase. Aggregate dissociation therefore does not provide a general explanation for enzyme activation. The structural features that make one enzyme more responsive than another remain unclear.

Treatment conditions introduce another source of variation in enzyme activation [14]. Cellulase activity increased progressively with ultrasonic power and reached a maximum enhancement of 18.17% at 15 W [93]. Beyond this level, cellulase activity declined. The authors attributed the decline to polypeptide-chain damage and changes in the conformation of the active site. Frequency also produced a non-linear response. Cellulase activity was enhanced at frequencies ranging from 18 to 26 kHz, but slightly decreased at 29 kHz [93]. The decline at 29 kHz was attributed to cavitation-induced heating and radical formation, although the relative contributions of these effects were not quantified independently. Pectinase activity likewise increased initially and then declined with treatment duration, reaching its maximum after 15 min; complete inactivation was reported after 35 min of sonication [94].

These power-, frequency-, and time-dependent responses describe an enzyme-specific operating window rather than a transferable optimum. The parameters cannot be interpreted independently because their combination determines the acoustic energy delivered to the enzyme solution. Reactor geometry, treatment volume, duty cycle, and temperature control further influence the actual exposure received by the enzyme. Equipment that permits independent control of frequency, power, and duty cycle would facilitate optimization, but wider adjustability alone would not make results comparable. Studies also need to report how absorbed acoustic energy was estimated and specify the treated mass or volume. Temperature history, reactor configuration, and enzyme activity should be documented using consistent methods. A further practical question is whether the activity gain persists during storage and subsequent hydrolysis, since most studies emphasize activity measured immediately after sonication.

### 5.3. Applications of Ultrasound to the Enzymatic Hydrolysis Process

Applying ultrasound during enzymatic hydrolysis differs from pretreatment because acoustic exposure continues while substrate conversion is underway. The enzyme, substrate, and reaction products are therefore sonicated within the same medium. Some studies use this approach primarily to increase hydrolysis efficiency, whereas others evaluate whether it also changes the structure and functionality of the resulting products. Representative findings are summarized in Table 2.

During hydrolysis, ultrasound can disrupt compact or aggregated substrates and expose cleavage sites that were previously inaccessible to the enzyme. It also maintains dispersion and renews the interface between the enzyme and the substrate as the reaction proceeds. Reported responses include increases in the degree of hydrolysis, soluble protein content, or peptide yield, depending on the substrate and the measurement used. In zein hydrolysis, ultrasound loosened the protein structure and increased the accessibility of cleavage sites, thereby promoting protein degradation [95]. This case mainly reflects improved substrate accessibility. The importance of mass transfer becomes clearer at high substrate concentrations. In a concentrated feather-protein system, ultrasound increased peptide yield by 6.08% and protein conversion by 18.63% [96]. By maintaining mixing during the reaction, in-process sonication can reduce transport resistance that may reappear after substrate pretreatment. This mode of application may therefore be useful in concentrated or viscous systems, where ordinary agitation provides insufficient contact between the enzyme and substrate. The benefits of improved mass transfer can be offset by enzyme inactivation during sonication. In soybean oil hydrolysis catalyzed by phospholipase, direct sonication increased the initial rate but reduced the final product yield. Emulsifying the substrate with ultrasound before enzyme addition gave better results [97]. This comparison suggests that ultrasound pretreatment of the substrate may be preferable to treatment during hydrolysis when enzyme damage outweighs the benefits of improved mixing.

In-process sonication has also been used when product properties are evaluated alongside conversion efficiency. In fava bean protein systems, dual-frequency ultrasound combined with enzymolysis induced structural unfolding and increased molecular flexibility. The resulting hydrolysates showed improved techno-functional properties and digestibility, supporting their potential use as functional food ingredients [98]. Ultrasound-assisted enzymatic hydrolysis has also been applied to resistant starch production. The resulting starch showed greater resistance to digestion and improved thermal stability compared with conventionally hydrolyzed products [99]. These protein and starch studies describe different forms of product modification rather than a single response to ultrasound. For peptides, the outcome depends on the molecular-weight distribution, sequence, and surface properties of the hydrolysate. For starch, changes in molecular organization and crystalline structure determine digestion and thermal behavior. Product-specific measurements are therefore required to establish whether ultrasound produces the properties needed for a particular food application. Most studies characterize hydrolysates immediately after processing, and their performance during food formulation, storage, and digestion is not evaluated consistently. Improved interfacial behavior or digestion resistance under laboratory conditions does not necessarily guarantee comparable performance in a complex food matrix. Future studies should assess conversion efficiency together with product stability and application-specific functionality. Such evaluation would show whether the additional acoustic treatment produces a useful product modification rather than only a short-term change in analytical indicators.

### 5.4. Mechanisms of Ultrasound-Assisted Enzymatic Hydrolysis

**Figure 4:**
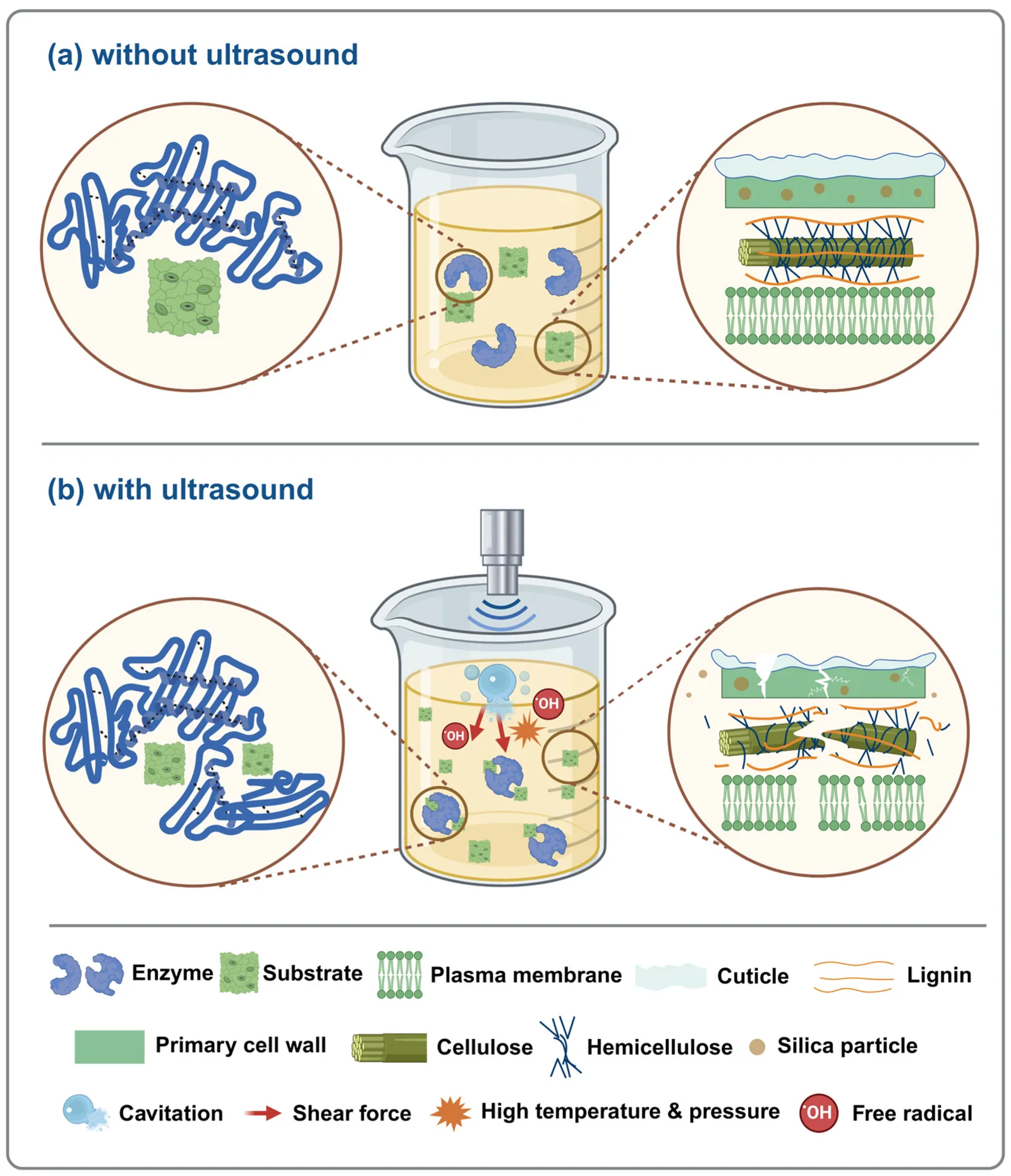
Schematic mechanisms of ultrasound-assisted enzymatic hydrolysis. (**a**) Without ultrasound, enzyme aggregation and intact substrate barriers may limit enzyme access. (**b**) Ultrasound disperses enzyme aggregates, alters enzyme conformation, and disrupts the substrate matrix, including the lignin–hemicellulose network, to expose cellulose. Cavitation-induced mixing may further improve mass transfer and enzyme–substrate contact.

The effects of ultrasound on enzymatic hydrolysis arise from acoustic cavitation and its associated mechanical and chemical effects. Bubble oscillation and collapse generate microjets, shear forces, microstreaming, and shock waves, while water sonolysis produces free radicals. These effects act on three targets within the hydrolysis system: the substrate, the enzyme, and the reaction medium (Figure 4).

As described in Section 3.4, mechanical disruption restructures substrates by reducing particle size, dispersing aggregates, and loosening compact networks. Radical reactions may also alter lignin components and protein side chains, although their contribution is less clearly separated from mechanical effects. In lignocellulosic substrates, disruption of the lignin–hemicellulose network exposes cellulose chains to cellulase and increases saccharification efficiency [88,100]. In protein substrates, partial unfolding changes the accessibility of peptide bonds to proteases and may alter the resulting peptide profile. Such changes do not always increase the overall degree of hydrolysis. Ultrasound increased the ACE-inhibitory activity of rice protein hydrolysates without changing their degree of hydrolysis [101]. A similar separation between hydrolysis degree and peptide activity was observed for edible bird’s nest protein [89]. This response may arise because ultrasound changes the relative accessibility of cleavage sites and the distribution of released peptides rather than the total extent of cleavage. The degree of hydrolysis should therefore be considered together with peptide composition and application-specific activity.

Ultrasound-induced activation of free enzymes has been associated with structural changes at several scales. The mechanical effects of acoustic cavitation are a proposed explanation for these changes. At the macroscopic scale, microstreaming and shear forces weaken intermolecular interactions and dissociate large enzyme aggregates into smaller ones, raising enzyme dispersity and the fraction of catalytically active molecules. This has been observed directly. Atomic force microscopy showed that ultrasound broke up large tyrosinase aggregates [102], and the particle size and polydispersity index of cyclodextrin glycosyltransferase fell markedly after sonication [78]. At the tertiary-structure level, a proposed mechanism involves shear-induced conformational changes in the folded enzyme, with potential effects on active-site geometry and substrate accessibility [22]. Consistent with this, ultrasound altered the tertiary structure of α-amylase and glucoamylase and increased their catalytic efficiency [79]. At the secondary-structure level, sonication changes the exposure of surface residues, particularly the microenvironment of tryptophan and tyrosine, and shifts the relative proportions of α-helix, β-sheet, and random coil [92,103]. These shifts are consistent with changes in local rigidity and molecular flexibility, potentially favoring catalytically competent conformations. These structural changes across the three scales may contribute to improved reaction kinetics. When ultrasound was applied with Ca^2+^ to activate α-amylase, the Michaelis constant (K_m_) decreased by 9.13% and the maximum reaction velocity (V_max_) increased by 37.4% [104], indicating both greater substrate affinity and higher catalytic efficiency.

During in-process hydrolysis, ultrasound also modifies transport within the reaction medium [105]. Microstreaming improves mixing and reduces transport resistance between the enzyme and substrate. In heterogeneous systems, cavitation can thin the diffusion boundary layer surrounding solid particles and accelerate substrate transfer to enzyme-accessible surfaces [106]. It also disperses insoluble aggregates and renews the solid–liquid interface [107]. Enhanced convection may reduce local product accumulation near these interfaces, although it does not necessarily eliminate inhibition caused by products accumulated in the bulk medium. Transport enhancement becomes more relevant in concentrated or viscous systems [96].

## 6. Ultrasound-Assisted Extraction

In food biomanufacturing, extraction provides substrates for subsequent biotransformation or recovers ingredients and bioactive compounds from raw materials and processed products. Unlike fermentation and enzymatic hydrolysis, ultrasound-assisted extraction (UAE) is usually applied while the material is in contact with the solvent, making classification by application stage less informative. Because extraction behavior and product stability depend on the target compound, this section organizes UAE applications by compound class before discussing the underlying mechanisms. Table 3 summarizes the target compounds, solvent systems, reported ultrasound conditions, and main extraction outcomes.

### 6.1. Target Compounds of Ultrasound-Assisted Extraction

UAE has been applied to recover proteins, lipids, polysaccharides, and low-molecular-weight bioactive compounds from food materials and processing residues. Protein studies have mainly evaluated improvements in release and diffusion. Dual-frequency ultrasound at 20/28 kHz increased both the release rate constant and the diffusion coefficient of soluble sunflower-seed protein relative to the control [116]. The practical value of these improvements also depends on their contribution to the complete production process. In a pilot study of soybean processing, okara suspensions were treated continuously at 20–100 kg/h in a 0.8 L ultrasonic flow cell. Ultrasound increased protein recovery during okara treatment by approximately 4.2 percentage points [117]. Given the energy input and concerns over probe durability, the authors did not recommend the tested process for industrial use. Lipid extraction requires a balance between recovery and oxidative stability. Higher ultrasonic power increased walnut-oil yield, whereas prolonged sonication impaired its quality indices [17]. During lipid extraction from Pacific white shrimp cephalothorax, ultrasound also promoted oxidation. Nitrogen protection and tannic acid addition mitigated this deterioration [110]. For polysaccharides, extraction yield must be considered together with the molecular characteristics of the product. A combined ultrasound–enzyme treatment increased polysaccharide recovery from sugarcane peel and produced fractions with antioxidant activity [118]. In carrot pomace, ultrasound conditions affected both pectin yield and molecular architecture [15]. Whether such structural modification is desirable depends on the intended application of the polysaccharide.

Low-molecular-weight bioactive compounds present a stronger trade-off between extraction yield and structural preservation. Ultrasound has increased the recovery of phenolics, flavonoids, saponins, and thymol from plant materials [119]. Cavitation-generated radicals may also modify sensitive molecular structures and reduce the activity retained in the extract. In rose polyphenol extraction, most of the frequencies tested reduced antioxidant activity, whereas dual-frequency ultrasound at 20/28 kHz preserved it [120]. The authors attributed this response to frequency-dependent differences in hydroxyl-radical generation. Because radical formation is also affected by power, treatment time, dissolved gases, and reactor configuration, the observed response cannot be assigned to frequency alone.

Natural deep eutectic solvents (NADES) have been combined with ultrasound to improve solubilization and selectivity and, in some systems, preserve sensitive compounds. The hydrogen-bond network of NADES can strengthen solute-solvent interactions and improve the stability of some phenolic compounds, although direct evidence that their low free-water content suppresses cavitation-derived hydroxyl radicals remains limited [121]. High viscosity can restrict diffusion and weaken cavitation, although ultrasound partly compensates through microstreaming and localized heating. A choline chloride–oxalic acid NADES combined with ultrasound increased both the yield and antioxidant activity of polyphenols extracted from apple pomace [122]. A choline chloride–fructose–water NADES (ChCl: Fru: H_2_O) produced a total phenolic content 818% higher than that obtained with 60% ethanol and also improved bioaccessibility [115]. Solvent polarity influenced selectivity, with hydrophilic NADES favoring phenolics and hydrophobic NADES favoring carotenoids and chlorophyll [123]. High viscosity and empirical solvent formulation remain practical limitations. Recovery of purified compounds from NADES is also difficult, while direct use of the complete extract requires evaluation of solvent safety, sensory effects, and compatibility with the final food formulation.

### 6.2. Mechanisms of Ultrasound-Assisted Extraction

In UAE, the mechanical and chemical effects of cavitation act mainly on the source matrix and the matrix-solvent system. Mechanical effects weaken structural barriers and accelerate the transfer of released compounds into the solvent, whereas radical and thermal effects may alter their chemical stability.

The physical basis of substrate restructuring is shared with the processes discussed in Section 3.4 and Section 5.4. Near a solid surface, microjets, shear forces, and shock waves rupture cellular structures, erode particles, and increase the surface area exposed to the solvent [7,124]. The extraction-specific consequence is the release of intracellular or matrix-bound compounds into the surrounding liquid. In lipid-containing materials, mechanical disruption opens cell membranes and lipid-storage structures, allowing intracellular oils to enter the solvent. In plant tissues, damage to cell walls and membranes releases phenolics, flavonoids, and other metabolites [119]. Radical reactions may also alter cell-wall polymers, proteins, or other matrix components, although mechanical disruption usually dominates compound release. For structurally sensitive macromolecules, the same forces that improve accessibility may also change the molecular characteristics of the recovered product.

After release, target compounds must move from the matrix surface into the bulk solvent. Microstreaming and turbulence improve mixing, thin the diffusion boundary layer around solid particles, and promote solvent penetration into the matrix [7]. Solvent properties influence different parts of this process. Polarity influences target-compound solubility and extraction selectivity, whereas viscosity affects diffusion, resistance to microstreaming, and cavitation development [125]. Vapor pressure and surface tension affect bubble formation and collapse. These relationships are evident with natural deep eutectic solvents, whose high viscosity restricts diffusion and weakens cavitation. Ultrasound-induced microstreaming and localized heating can partly offset this limitation, consistent with the improved extraction of polyphenols from apple pomace [122]. Kinetic evidence is provided by dual-frequency ultrasound, which increased the release rate constant and diffusion coefficient of soluble sunflower-seed protein [116]. The contribution of ultrasound becomes more relevant when high viscosity or poor solvent penetration limits diffusion.

Once released into the solvent, target compounds remain exposed to the chemical and thermal effects of cavitation. Hydroxyl radicals formed during water sonolysis can initiate oxidation, while local heating may accelerate related reactions. In lipid-containing systems, these effects promote the oxidation of unsaturated fatty acids and can reduce oil quality [110]. In extracts containing phenolic compounds, radical reactions may oxidize phenolic hydroxyl groups and conjugated structures associated with antioxidant activity. This process may contribute to the loss of antioxidant activity observed under some ultrasound conditions [120]. The extent of chemical modification depends on acoustic exposure and on the solvent environment, including water content and oxygen availability. Most studies infer radical-mediated degradation from changes in oxidation indices or antioxidant activity rather than measuring radical formation directly. Radical-scavenging controls and matched thermal treatments are therefore needed to separate sonochemical modification from oxidation caused by bulk heating or oxygen exposure.

## 7. Integration of Ultrasound with Complementary Technologies

Ultrasound alone may provide insufficient structural disruption or mass transfer in complex food matrices, while greater acoustic exposure can damage sensitive components. Researchers have therefore combined ultrasound with other processing technologies to improve conversion or recovery within the limits imposed by product stability. The combinations examined here include pulsed electric fields, cold atmospheric plasma, microwave processing, and membrane separation. This section examines their mechanistic basis and reported applications, with attention to whether the combined treatment provides an advantage over either technology alone.

### 7.1. Pulsed Electric Fields

Pulsed electric fields (PEF) increase cell membrane permeability through electroporation, whereas ultrasound promotes structural disruption and mass transfer through acoustic cavitation. PEF pretreatment can make intracellular compounds more accessible before ultrasound facilitates solvent penetration and solute release. Applications have focused mainly on extraction and the valorization of food by-products. In rosemary and thyme residues, PEF pretreatment increased phenolic recovery during subsequent ultrasound-assisted extraction [126]. Combined PEF and ultrasound treatment also improved chickpea protein extraction and modified the functional properties of the resulting isolate [127]. The response was different in sunflower seeds, where simultaneous treatment produced less oil than either technology alone [128].

Applications in biological conversion are less developed than those in extraction. In liver co-fermentation, combined ultrasound–PEF treatment increased membrane permeability and substrate utilization, promoted protein degradation, and enhanced taste-active amino acid formation [129]. Both greater substrate accessibility and altered microbial activity may contribute to this response. Their relative contributions are difficult to distinguish when the substrate and microorganisms are exposed together. Comparison of substrate pretreatment with treatment after inoculation would help identify the main cause of improved conversion. Such comparisons should control temperature and measure microbial viability alongside product formation. Treatment conditions selected for extraction cannot be transferred directly to an active fermentation because irreversible membrane damage can increase compound release while impairing metabolism. During scale-up, differences in electric and acoustic exposure within the reactor may further alter the balance between stimulation and cell injury.

### 7.2. Cold Atmospheric Plasma

Cold atmospheric plasma (CAP) modifies food matrices through reactive species generated in an ionized gas. In extraction, surface modification by CAP can improve access for the solvent before ultrasound promotes compound release and transport. In *Capsicum annuum* waste, CAP pretreatment disrupted the surface structure and facilitated carotenoid release during subsequent sonication [130]. A similar sequence enhanced bioactive compound recovery from cornelian cherry pomace [131]. Combined ultrasound and CAP treatment also increased flavonoid and glycyrrhizic acid contents in liquorice root extracts relative to conventional maceration [132]. This comparison establishes an improvement over maceration, but it does not isolate the contribution of each treatment. Controls using CAP and ultrasound separately are needed to determine whether coupling provides an additional benefit.

CAP–ultrasound treatment can also modify macromolecular properties, as shown by changes in shrimp tropomyosin. Ultrasound-assisted CAP altered its structure and reduced IgE- and IgG-binding capacities by 52.40% and 46.51%, respectively [133]. These binding measurements indicate altered immunoreactivity but do not by themselves establish reduced allergenicity after consumption. The oxidative reactions involved in protein modification also require attention when the objective is to preserve sensitive compounds or biocatalysts. In fermentation and enzyme-catalyzed processing, reactive species may affect the substrate and the microorganisms or enzymes needed for conversion. Treatment before inoculation or enzyme addition could separate matrix modification from direct biocatalyst exposure. Measuring product formation together with oxidation and biocatalyst activity would help resolve this balance.

### 7.3. Microwave Processing

Microwave (MW) processing produces dielectric heating, whereas ultrasound disrupts structures and enhances transport through cavitation. This combination has been investigated mainly for extracting bioactive compounds from food materials and processing residues. Ultrasound–MW-assisted extraction increased the yield and in vitro antioxidant activity of *Dictyophora indusiata* polysaccharides compared with either treatment alone [134]. Recovery and structural preservation did not coincide during alginate extraction from *Nizamuddinia zanardinii* by-products. MW treatment gave the highest yield, while the combined treatment caused a greater reduction in molecular weight [135]. Greater depolymerization may be useful for some applications but undesirable when viscosity or gel formation depends on retaining long polymer chains. Extraction yield should therefore be evaluated together with the molecular properties required in the final ingredient.

Ultrasound–MW treatment has also been investigated in enzymatic processing. In sweet potato protein, the combined treatment increased the degree of hydrolysis. The resulting peptide fractions showed higher antioxidant activities than those obtained without ultrasound–MW treatment, with strong activity in fractions below 3 kDa [136]. In vegetable residues, ultrasound- and MW-assisted treatment with a ternary deep eutectic solvent removed more than 90% of lignin from garlic skin and green onion roots. This enriched the cellulose fraction and improved its accessibility to subsequent cellulase hydrolysis [137]. The solvent contributes to delignification in this combined pretreatment, so the improvement cannot be assigned to the two physical treatments alone.

Heating complicates interpretation of ultrasound–MW effects because faster extraction or hydrolysis may result from a higher temperature rather than an interaction between the fields. A conventional thermal control should reproduce the treatment’s time–temperature history, while comparisons with each technology alone should account for energy input. Measurements of molecular degradation are also needed when greater recovery is accompanied by depolymerization. Direct evidence in fermentation and aging remains sparse. In fermentation, temperature must remain compatible with the microorganisms, whereas aging requires control of heat-induced flavor changes. Reactor scale-up adds a spatial constraint because microwave heating and cavitation may be distributed unevenly. Their overlap should be assessed to identify regions of excessive heating or insufficient treatment.

### 7.4. Membrane Separation

Membrane separation fractionates or concentrates food components, but concentration polarization and fouling reduce flux during operation. Ultrasound applied near the membrane generates microstreaming and shear that disturb the boundary layer and limit foulant deposition. In cross-flow ultrafiltration of skim milk, simultaneous ultrasound disrupted the concentrated layer and increased permeate flux without changing casein-micelle structure or membrane selectivity [138]. Microfiltration combined with ultrasonication also extended refrigerated skim-milk shelf life to at least 40 days and retained more bioactive proteins than thermal pasteurization [139]. This result concerns microbial control and protein retention within a combined process; it should be distinguished from direct evidence of improved membrane transport.

The position of ultrasound within the process determines how it contributes to downstream recovery. Ultrasound-assisted extraction followed by ultrafiltration improved saccharide separation from *Arthrospira platensis*, although membrane fouling remained detectable [140]. Here, ultrasound changes the feed entering the membrane rather than acting on the membrane surface during filtration. During nanofiltration concentration of *Ganoderma lucidum* glycopeptides, ultrasound reduced concentration polarization and improved separation efficiency [141]. Ultrasound-assisted gradient dilution feeding has also been integrated with ultrafiltration during chicken-protein hydrolysis. It increased peptide yield and umami intensity while reducing substrate accumulation associated with fouling [142]. These configurations should be compared according to the step being intensified, since improved extraction, hydrolysis, and membrane transport are distinct contributions.

For ultrasound applied during filtration, the operating objective is to sustain transport while preserving separation selectivity and membrane integrity. Higher flux alone is insufficient if membrane damage allows unwanted compounds to pass through or changes the retained product. Localized or pulsed ultrasound may limit bulk-product exposure while acting on deposits near the membrane. Longer operating trials should relate flux recovery and cleaning frequency to energy use, membrane lifetime, and product quality.

## 8. Emerging Food Biomanufacturing Platforms and Opportunities for Power Ultrasound

Food biomanufacturing increasingly uses engineered microorganisms, selected microbial communities, and cell-free enzyme systems to produce defined food ingredients. These platforms differ in how biological activity is organized and in what constitutes the final product. Precision fermentation uses an engineered strain to synthesize a specific molecule, whereas biomass fermentation produces microbial cells as the main source of protein. Synthetic microbial communities distribute metabolic functions among interacting strains, while cell-free systems perform conversion outside living cells through enzymes or crude lysates. These differences determine where ultrasound could be introduced and which biological component is most vulnerable to acoustic exposure. Table 4 summarizes the current evidence and future directions for power ultrasound in emerging food biomanufacturing platforms.

### 8.1. Precision Fermentation and Microbial Cell Factories

Precision fermentation uses selected or genetically engineered microorganisms as cell factories for the production of specific food ingredients. The target is a defined molecule rather than the fermented matrix or microbial biomass itself. Current products include recombinant dairy and egg proteins, human milk oligosaccharides, enzymes, pigments, flavor compounds, vitamins, and functional lipids [154]. For example, *Trichoderma reesei* has been engineered to produce bovine β-lactoglobulin and hen egg ovalbumin with relevant food-processing properties [143]. Engineered *Escherichia coli* strains have also been developed for the selective synthesis of structurally complex human milk oligosaccharides such as lacto-N-fucopentaose I [144]. In these systems, productivity depends on the metabolic capacity of the host, the expression and secretion of the target molecule, and the supply of oxygen and nutrients during high-density cultivation.

Direct evidence for ultrasound in precision fermentation is still limited. A related study applied low-intensity ultrasound during ε-polylysine fermentation by *Streptomyces albulus*. Ultrasound altered intracellular ATP levels and the activities of enzymes involved in ε-polylysine biosynthesis, with a corresponding increase in product formation [145]. This result shows that acoustic treatment can affect the metabolic state of a production strain. It does not establish that the same treatment would improve recombinant protein or oligosaccharide production. *S. albulus* is a native metabolite producer rather than a genetically engineered precision-fermentation host, and recombinant strains may respond differently because heterologous expression already imposes metabolic and secretory stress.

The appropriate point of ultrasound application should therefore be selected according to the limitation of each production system. Medium pretreatment may improve the accessibility of complex feedstocks without exposing the engineered cells to cavitation. During cultivation, low-intensity ultrasound could assist mixing or oxygen transfer when transport restricts productivity, but direct cellular exposure may compromise membrane integrity, plasmid stability, protein folding, or secretion. Intracellular products present a different opportunity. Ultrasound can be applied after cultivation to disrupt harvested cells, where loss of viability is no longer detrimental and selective product release becomes the main objective.

### 8.2. Biomass Fermentation for Microbial Protein Production

In biomass fermentation, the microbial cells constitute the principal product. Yeasts, filamentous fungi, bacteria, and microalgae can convert sugars, agricultural side streams, or other carbon sources into protein-rich biomass. This route is being investigated as a means of producing food protein with less dependence on arable land and animal agriculture [155]. Some platforms use agro-industrial by-products as substrates, while others rely on purified sugars or gaseous carbon sources. The process commonly includes substrate preparation, biomass cultivation, harvesting, cell disruption, protein recovery, and functional modification.

This production sequence offers several points for ultrasound intervention, but the intended effect changes with treatment stage and acoustic dose. During cultivation, mild ultrasound may reversibly increase membrane permeability and improve substrate dispersion, oxygen transfer, and nutrient transport, thereby supporting cell growth and biomass accumulation. Stronger or prolonged exposure can convert this reversible response into membrane damage or cell lysis, reducing viable biomass and yield. Direct evidence from microbial-protein fermentation remains limited, so operating windows established in conventional fermentations cannot be transferred without validation. After harvesting, cell disruption becomes desirable because intracellular proteins must be released from resistant cell walls. Membrane disruption therefore shifts from a biological risk during cultivation to a useful recovery mechanism after biomass harvest.

Evidence for this post-harvest use is stronger than that for sonication during cultivation. Ultrasound released protein and polysaccharide from yeast cells, with selectivity governed by treatment intensity and duration [146]. It also disrupted fungal mycelia and improved the recovery of polysaccharide–protein complexes from a viscous fermentation broth [147]. These findings are relevant to fungal and yeast protein production because incomplete cell disruption can limit protein recovery and digestibility. Ultrasound may also modify the recovered protein after release. When combined with pH shifting, ultrasonication changed the structure, functional properties, digestibility, and bioactivity of yeast protein [148]. The outcome cannot be judged from extraction yield alone because structural modification may improve solubility or interfacial performance while reducing other properties required for a particular food formulation.

### 8.3. Synthetic Microbial Communities for Directed Food Fermentation

Synthetic microbial communities are assembled from selected strains whose metabolic activities complement one another. They offer greater control than spontaneous fermentation while retaining functions that are difficult to reproduce with a single microorganism. Individual strains may divide the conversion of substrates, exchange metabolites, remove inhibitory compounds, or contribute different aroma and texture attributes. Research on food-associated synthetic communities has focused on community resistance, resilience, and functional stability [149]. A recent example used a designed microbial community to increase the formation of characteristic flavor compounds during sesame-flavor Baijiu fermentation [150].

Ultrasound has rarely been examined directly in synthetic microbial communities. Studies of single strains show that pretreatment can alter microbial growth, enzyme activity, and fermentation performance [47], but such responses cannot be extrapolated directly to multispecies cultures. Ultrasound applied to the substrate before inoculation may release nutrients or change its rheological properties, thereby modifying the environment in which the community subsequently develops. Because no microorganisms are present during this treatment, differences in strain sensitivity do not directly affect community composition. Treating the inoculum or an active fermentation is different. All strains are exposed simultaneously, but their acoustic tolerances may vary, creating unintended selection pressure. Community composition could therefore shift away from its designed state even if total biomass or product concentration increases. Detecting such changes requires measurements beyond final flavor concentration and fermentation rate. Future studies should combine strain-abundance measurements with metabolite profiling and assess whether functional redundancy and community stability are preserved after treatment.

### 8.4. Cell-Free and Multi-Enzyme Biomanufacturing

Cell-free biomanufacturing performs biological conversion outside intact living cells. Reactions may use crude cell lysates, purified enzymes, or organized multi-enzyme cascades [156]. Removing the cell boundary allows direct control over enzyme composition, substrate concentration, and reaction conditions. It also avoids constraints associated with cell viability, membrane transport, and the toxicity of substrates or products. Multi-enzyme systems can combine sequential reactions, cofactor regeneration, and selective molecular modification to produce food ingredients that would be difficult to obtain through a single hydrolytic step.

The distinction from conventional enzymatic hydrolysis lies in the process objective. Hydrolysis mainly depolymerizes proteins or carbohydrates, whereas cell-free manufacturing often directs synthesis or selective transformation along a designed pathway. Engineered protein scaffolds have been used to assemble enzymes and improve flux through cell-free biosynthetic systems [151]. Cascade biocatalysis has also been combined with host engineering and substrate-feeding strategies for the synthesis of the high-intensity sweetener rebaudioside M [157]. In these systems, performance depends on the coordination of several catalysts rather than the maximum activity of one enzyme.

Ultrasound could enter a cell-free process during lysate preparation, enzyme formulation, immobilization, or reaction. Sonication is already used to disrupt microbial cells for lysate production, but the delivered energy must be controlled. Cell-free expression in *E. coli* lysates showed a complex dependence on the sonication energy used during lysis, indicating that greater disruption does not necessarily produce a more active lysate [152]. Excessive treatment may damage enzymes, ribosomes, nucleic acids, or cofactors even when cell breakage becomes more complete.

Evidence from food-enzyme research supports additional uses. Mild ultrasound can disperse enzyme aggregates and alter enzyme conformation, sometimes increasing catalytic activity [22]. Ultrasound-assisted biocrosslinking has also been used to improve enzyme immobilization [153]. These effects could benefit cell-free cascades by increasing catalyst accessibility or reducing diffusional resistance. Systems containing insoluble substrates may gain from continuous interface renewal, while immobilized cascades may benefit from improved transport through the carrier structure.

The response of an individual enzyme does not predict the performance of an entire cascade. Increasing the activity of one catalyst may simply move the rate limitation to the next reaction. Different enzymes may also have incompatible acoustic tolerances, and cofactors can become unstable under conditions that leave the protein catalysts largely intact. The relevant outcomes are therefore pathway flux, product selectivity, cofactor turnover, and operational stability rather than the activity of one isolated enzyme.

## 9. Challenges and Future Perspectives

### 9.1. Current Challenges

As summarized in Table 5, ultrasound can improve bioconversion and target compound recovery, shorten processing time, and enhance selected product properties. These benefits are not observed in every system. Some studies found no significant increases in biomass, degree of hydrolysis, or extraction yield, indicating that structural or physiological changes do not necessarily improve process performance. Others reported reduced enzyme activity, pigment loss, diminished bioactivity, or lipid oxidation. Benefits and adverse effects can occur within the same treatment, so higher yields or faster reactions do not necessarily lead to better product quality. Ultrasound performance should therefore not be judged solely by the largest improvement in a single indicator under optimized conditions. Assessment should also consider whether other important properties remain stable and whether greater treatment intensity causes additional damage. Differences in treatment conditions, controls, and evaluation criteria further limit direct comparisons among studies. Wider application requires a clearer distinction between beneficial and damaging exposure, reproducible treatment, and evidence that laboratory improvements translate into industrial benefits. These goals remain constrained by limited mechanistic understanding and predictive capability, difficulties in acoustic-field delivery and process control, and insufficient scale-up studies and industrial validation.

#### 9.1.1. Mechanistic Understanding and Predictive Capability

Current interpretations of cavitation effects rely mainly on observed changes in processing outcomes. Quantitative relationships between absorbed acoustic energy and target responses remain insufficiently characterized across different food systems. This gap is difficult to address in complex foods because molecular changes cannot yet be linked reliably to reaction kinetics. Matrix and solvent properties further influence cavitation, but predictive models seldom account for them. Many food materials scatter or attenuate sound because they are heterogeneous or viscous. Studies also describe acoustic exposure using incompatible measures. Some report nominal power or amplitude, whereas others use power density or treatment time. These differences hinder comparison and leave processing conditions dependent on empirical optimization. Models that connect reactor design with product response are therefore still lacking.

#### 9.1.2. Acoustic-Field Delivery and Process Control

Transducer output and frequency may drift during prolonged operation. A more immediate problem is the uneven distribution of acoustic energy. Cavitation is concentrated near the transducer and weakens with distance, producing spatial differences in treatment intensity and product response. Nominal equipment settings provide little information about the energy received in different regions of the material. This uncertainty is important for enzymes and microorganisms because the range between stimulation and inactivation can be narrow. Cavitation also changes as the product heats, loses moisture, or becomes more viscous. Variation in dissolved gas introduces another source of instability. Fixed power and treatment time therefore do not guarantee constant acoustic exposure.

#### 9.1.3. Scale-Up and Industrial Validation

The required ultrasound conditions depend on the material and processing objective, making a universal treatment protocol neither feasible nor necessary. Standardization should instead focus on how absorbed acoustic energy is measured and calculated and how reactor geometry is characterized. Treatment volume and temperature history must also be documented. Product validation presents a separate challenge. Safety and stability during storage have not been examined consistently, while sensory and nutritional limits remain product-specific. Most studies use laboratory-scale volumes. Simply maintaining the same nominal power density during scale-up does not reproduce the original cavitation field because reactor geometry changes. Industrial feasibility depends on both energy use and processing capacity. These costs must be weighed against gains in yield, shorter processing times, or reduced solvent consumption. Without energy-normalized and throughput-based comparisons, it remains unclear whether laboratory improvements provide a net benefit under continuous production conditions.

### 9.2. Future Perspectives

Future research should address how ultrasound conditions can be selected for new systems, maintained during processing, and validated for industrial use. The following sections discuss mechanism-based prediction to guide parameter selection, acoustic-field control to support reproducible treatment, and industrial validation to assess technical feasibility, economic viability, and environmental impacts.

#### 9.2.1. Mechanism-Based Prediction

Future research should combine existing evidence with mechanistic understanding to guide ultrasound parameter selection for new food materials and processes. A database linking material properties, processing objectives, treatment conditions, and reported outcomes could help identify relevant studies and initial parameter ranges. Records should include ineffective treatments and adverse effects to help define the limits of ultrasound application. Mechanistic findings could then help predict how ultrasound affects heat and mass transfer and the structure of components in new food biomanufacturing systems. For example, high-speed imaging can characterize bubble dynamics in optically accessible systems, while molecular simulations can examine solvent–solute interactions and their effects on extraction selectivity. These investigations should link physical or molecular changes to processing outcomes across different acoustic exposures, helping distinguish beneficial modifications from damage. The resulting predictions should be tested in new materials, with experimental results used to refine the proposed parameter ranges and update the database.

#### 9.2.2. Acoustic-Field Control

Acoustic-field control should aim to improve spatial uniformity, consistency across processing scales, and long-term operational stability. Spatial uniformity concerns differences in acoustic intensity within the treatment volume, including those associated with distance from transducers and standing-wave patterns. Distributed transducers and reactor-geometry optimization may reduce the strong spatial gradients produced by a single probe. Future research should evaluate chamber dimensions, reflecting-surface geometry, and transducer placement, with multifrequency or sweeping-frequency operation explored as complementary approaches. Consistency across scales means maintaining comparable acoustic exposure when treatment volume changes. Recirculating systems with adjustable numbers of transducers and parallel modular units should be investigated for this purpose. Closed-loop ultrasonic reactors could adjust their output as temperature, viscosity, moisture content, or dissolved gas changes during processing. Combining acoustic monitoring with measurements of target-product properties could help guide these adjustments. Long-term stability concerns the ability to maintain acoustic performance. Future development should improve generator reliability, transducer durability, and bonding methods to limit power decline, frequency drift, and transducer detachment. These improvements should be evaluated during prolonged operation and repeated cleaning cycles.

#### 9.2.3. Industrial Validation

Industrial application of ultrasound requires technical feasibility testing, techno-economic analysis (TEA), and life cycle assessment (LCA). Laboratory treatment conditions should not be transferred directly to large-scale production. Parameters should be refined through progressive scale-up and pilot trials, with particular attention to acoustic-field distribution in the new reactor. These trials should also evaluate batch-to-batch product consistency and storage stability to establish technical feasibility. TEA should assess economic feasibility by relating process performance to capital investment, operating costs, and potential economic returns. This assessment should use measured electrical energy consumption per unit of product and account for equipment utilization and maintenance requirements. LCA should determine whether ultrasound reduces the environmental burden of the complete processing route. Comparisons need a common functional unit, such as one kilogram of product meeting defined specifications, and consistent system boundaries. In extraction, for example, solvent savings should be evaluated alongside energy demands for sonication, cooling, and solvent recovery. This would reveal whether a benefit at the extraction stage is offset elsewhere. Combined treatments should also be compared with each technology alone to assess the consequences of the additional processing step.

## 10. Conclusions

Power ultrasound has been applied at different stages of food biomanufacturing. Its effects depend on the treatment stage, process limitation, acoustic exposure, and stability of the treated material. Mild exposure may stimulate microorganisms or enzymes, whereas stronger treatment is better suited to cell disruption and the opening of recalcitrant matrices. Combining ultrasound with other technologies offers opportunities to exploit their complementary strengths, although synergistic effects require verification. Emerging food biomanufacturing platforms also warrant attention as potential areas for expanding ultrasound applications. Overall, the value of ultrasound lies in addressing the main limitation of each process without compromising product quality. Industrial translation will require reproducible control of acoustic exposure, consistent product performance, and sufficient processing gains to justify electrical energy consumption and equipment costs.

## Figures and Tables

**Table 1:** Applications of ultrasound at different stages of fermentation.

Ultrasound Treatment Stage	Substrates	Strains	Reported Optimal Conditions	Main Result	References
Substrate	Seaweed	Symbiotic culture of bacteria and yeast	25 kHz, 550 W (RP ^1^), 30 min	Increased organic acid content, acid-soluble protein content and volatile compounds; Reduced allergen content and pH.	[24]
	Chicken liver	*Pediococcus pentosaceus*	560 W (RP), 30 min	Decreased crude protein content; Increased amino acid nitrogen content, free amino acids, hydrolyzed amino acids and protein degradation rate.	[25]
	White finger millet	*Lactobacillus rhamnosus*	20 kHz, 0.99 W/mm^2^ (USI ^2^), 11.09 min	Reduced fermentation time; Promoted microbial proliferation and increased lactic acid production rate; Altered the apparent viscosity and water retention of the beverage.	[26]
	Wheat bran	*Aspergillus niger *ATCC-6275	20 kHz, 5 min	Promoted the degradation of polysaccharides and increased carbohydrate content; Increased the total amount of phenolic compounds; Enhanced in vitro antioxidant activity.	[27]
	*Citrus reticulata* peel	*Aspergillus niger *CGMCC 3.6189	40 kHz, 210 W (RP), 30 s-on/30 s-off, 28 °C, 39 min	Altered the structure and color of orange peel. Improved antioxidant activity;	[28]
Strain	*Citrus reticulata* peel	*Aspergillus niger *CGMCC 3.6189	20/40/60 kHz (simultaneous), 270 W (RP), 10 min, 30 s-on/30 s-off, 30 °C	Applied ultrasound at the logarithmic metaphase; Enhanced key enzyme activity and promoted flavonoid biosynthesis; Increased total soluble protein and carotenoid content.	[28]
	Rice wine	*S. cerevisiae *1048	28 kHz, 45 W/L (PD ^3^)	Applied ultrasound at the logarithmic metaphase; Enhanced the biomass and ethanol tolerance of strain; Enhanced intracellular and extracellular mass transfer.	[12]
	Jujube juice	*L. plantarum *CICC 20022	324 W (RP), 20 min, 1 s-on/4 s-off	Applied ultrasound at the logarithmic metaphase; Enhanced the activities of H^+^-ATPase and β-glucosidase; Increased flavonoid content, acidity and antioxidant capacity; Decreased pH and sugar concentration.	[29]
	Soybean meal	*Bacillus subtilis *10160	28 kHz, 140 W (RP), 1.5 h	Applied ultrasound at logarithmic prophase; Increased biomass, cell membrane permeability, metabolic capacity and peptide content.	[1]
	Apple juice	*Lactobacillus plantarum*	20 kHz, 58.3 W/L (PD), 30 min, 30 °C	Applied ultrasound at lag and logarithmic phases; Increased microbial count and lactic acid content; The metabolism of organic acids and free amino acids was correlated with phenolic metabolism.	[30]
Process	Soybean protein	*Limosilactobacillus fermentum *SCUT221015	20 kHz, 150 W (RP), 20 min, 2 s-on/2 s-off	Increased pyrazine, esters, and sulfur compounds, reduced undesirable aldehydes, and adjusted volatile characteristics; Promoted the production of 11 key aromatic active compounds; Enhanced aroma complexity and umami intensity.	[31]
	Ginkgo kernel juice	*Lactiplantibacillus plantarum*	20 kHz, 173.88 W/L (PD), 6 min, 5 s-on/5 s-off, 30 °C	Increased cell number and membrane permeability; Promoted the metabolism of phenolic acids and enhanced antioxidant activity in vitro; Reduced 4′-O-methyl pyridoxine toxin.	[32]
	Yogurt	*L. delbrueckii subsp. bulgaricus* and *S. thermophilus *(YF-L904)	28 kHz, 40 W/L (PD), 6 min, 100 s-on/10 s-off, 42 °C	Promoted microbial proliferation; Reduced fermentation time; Improved water retention of yogurt.	[33]
	Coffee leaf kombucha	Symbiotic culture of bacteria and yeast	40 kHz, 8 W/L (PD), 20 min, 30 s-on/30 s-off, 37 °C	Enhanced antioxidant activity; Enhanced α-glucosidase inhibitory activity.	[34]
	Doughs	Yeast	40 kHz, 300 W (RP), 40 min, 60 ms-on/40 ms-off, 30 °C	Enhanced the cross-linking between gluten and soy protein fractions, improving the stability of the gluten network; Promoted the aggregation of 7S/11S globulin with gluten, enhancing dough quality.	[35]

^1^ Reported power with the basis not specified in the original study. ^2^ Ultrasound intensity. ^3^ Power density.

**Table 2:** Applications of ultrasound at different stages of enzymatic hydrolysis.

Ultrasound Treatment Stage	Substrates	Enzymes	Reported Optimal Conditions	Main Result	References
Substrate	Brewer’s spent grains	Cellulase enzyme	20 kHz, 70.6 W/dm^3^ (PD ^1^), 30 min, room temperature	Increased hydrolysable cellulose content in the substrate; Increased glucose content in fermentation product.	[74]
	Oyster protein	Pepsin and trypsin	20 kHz, 200 W (RP ^2^), 20 min, 2 s-on/2 s-off, 0 °C	Increased soluble peptide content and antioxidant activity; Increased exposure of hydrophobic regions, promoting protein structure unfolding; Alleviated H_2_O_2_-induced oxidative stress and osteogenic dysfunction in MC3T3-E1 cells.	[2]
	Egg yolk powder	Neutral, alkaline and flavor enzymes	65 W (RP), 50 min, 2 s-on/2 s-off	Increased oil extraction rate, soluble protein content, and peptide content; Enhanced activities of lowering uric acid, blood sugar, and weight loss peptides; Altered the secondary structure of proteins, increasing the exposure of hydrophobic regions.	[75]
	*Cordyceps militaris* medium residues	α-Amylase and alkaline protease	20/28 kHz (sequential), 130 W/L (PD), 50 min, 50 °C	Increased ACE inhibitory activity; Established an in situ detection model for ACE inhibitory activity.	[76]
	Porcine bone collagen	Alkaline	450 W (RP), 20 min	Increased peptide content; Enhanced antioxidant and anti-inflammatory activities of the product; Altered protein secondary structure, increasing α-helix content and reducing β-sheet and random coil structures.	[77]
Enzymes	Pumpkin seed proteins	Neutrase, Brauzyn and Flavourzyme	40 kHz, 23.8 W/L (PD), 90/60/90 min, 25 °C	Activated Neutrase (22.7%), Brauzyn (20.8%), and Flavourzyme (22.4%); Altered the tertiary structure of the enzyme, increased particle size and polydispersity index; Enhanced the degree of hydrolysis, peptide content, and antioxidant activity.	[14]
	Maltodextrin	Cyclodextrin glycosyltransferase	20 kHz, 38 W/L (PD), 30 min, 25 °C	Increased enzyme activity (by 93%); Increased intrinsic fluorescence intensity; Decreased particle size and polydispersity index.	[78]
	Starch	α-Amylase and glucoamylase	40 kHz, 30/60 W (RP), 5/3 min, 25 °C	Activated α-Amylase (17.82%) and glucoamylase (38.5%); Expanded optimal temperature ranges of enzymes; Increased V_max_ and Catalytic efficiency; Changed the secondary and tertiary structures.	[79]
	*p*-nitrophenyl acetate	Lipase	22 kHz, 11.38 W/cm^2^ (USI ^3^), 25 min, 25 °C	Increased lipase activity (by 1.6 times); The thermal stability of lipase embedded in MOF was improved by 3.2 times.	[80]
	Goat cream	Lipase	25 kHz, 22 W/L (PD), 40 min, 25 °C	Increased lipase activity (by 12%); Enhanced fat hydrolysis rate.	[81]
Process	Potato protein	Alcalase, neutrase and flavourzyme	2% amplitude, 40 W/L (PD), 3 h, 5 s-on/5 s-off	Ultrasound probes were more effective than ultrasound baths; Increased hydrolysis degree and the antioxidant activity of peptides; Peptides with molecular weights between 1–2 kDa and less than 1 kDa exhibited higher antioxidant activity; Hydrophobic amino acids at the N-terminus and C-terminus enhanced the radical scavenging ability.	[82]
	Housefly larvae protein	Alcalase	20 ± 2/28 ± 2 kHz (simultaneous sweeping), 42 W/L (PD), 25 min, 5 s-on/1 s-off	Increased peptide yield and DPPH scavenging capacity; Improved solubility, foaming and emulsification properties of polypeptides; Increased α-helix, β-turn, and random coil, decreased β-sheet.	[83]
	*Zophobas morio* protein	Alcalase	100 kHz, 600 W (RP), 1 h, 50 °C	Increased hydrolysis degree; Changed secondary structure, with a decrease in α-helix and β-sheet, and an increase in β-turn and random coil; Increased antioxidant activity.	[84]
	Rice starch	α-Amylase and glucoamylase	22 kHz, 450 W (RP), 25 min, 2 s-on/2 s-off, room temperature	The ultrasonic thermal effect significantly enhanced the pore formation and adsorption properties of porous starch; The ultrasonic thermal effect primarily acted on the amorphous region of starch, with no significant impact on the A-type crystal structure; Enhanced the thermal stability, solubility, and swelling capacity of porous starch.	[85]
	Food waste	Amyloglucosidase enzyme	35 kHz, 35 W (RP), 10 h, 2 min-on/8 min-off, 30 °C	Shortened hydrolysis time (from 42 h to 10 h); Increased hydrolyzed sugar content; Decreased α-helix and increased random coil; Structural changes widened enzyme binding pockets, facilitating easier substrate access and product transport.	[86]

^1^ Power density. ^2^ Reported power with the basis not specified in the original study. ^3^ Ultrasound intensity.

**Table 3:** Applications of ultrasound-assisted extraction.

Substrates	Target Compounds	Solvent	Reported Optimal Conditions	Main Result	References
Cottonseed meal	Protein	4.6 g/L NaOH	320 W (RP ^1^), 53 °C, 21 min	Increased protein extraction yield to 58.23%; Achieved 93.21% free-gossypol removal and 87.56% total-gossypol removal; Induced partial protein unfolding and reduced molecular weight.	[108]
Amur grape seed	Oil	n-hexane	28/33 kHz (multi-frequency), 25 W/L (PD ^2^), 30 min	Improved the oil yield and oil quality; Enhanced anthocyanin content; Established an in situ real-time monitoring model.	[109]
Cephalothorax of Pacific white shrimp	Lipids	Mixture of isopropanol/n-hexane (1:1)	Ultrasonic amplitude: 91.2 µm, 25 min	Increased lipid yield; Reduced lipid oxidation through nitrogen flushing; Improved astaxanthin retention and polyunsaturated fatty acid stability.	[110]
Jujube	Polysaccharides	Water with added pectinase	22/33 kHz (multi-frequency), 300 W (RP), 30 min, 60 °C	Increased polysaccharides yield and antioxidant activity; Increased key monosaccharide content; Reduced molecular weight and viscosity.	[111]
*Acanthus ilicifolius*	Polysaccharides	Water	20 kHz, 150 W (RP), 53 °C, 50 min	Improved the extraction yield of *A. ilicifolius* polysaccharides; Altered the polysaccharide structure and reduced molecular weight; Enhanced the in vitro antioxidant activity of the extracted polysaccharides.	[112]
*Flammulina velutipes* stem waste	Bioactive polyphenols	75% ethanol (added nonionic surfactant Genapol-X080)	160 W (RP), 90 min, 44 °C	Enhanced the extraction rate by 27.35%; Decreased energy use by 66.67%; Increased antioxidant activity of the product.	[113]
*Sophora japonica*	Bioactive compounds	water or 70% ethanol	600 W (RP), 15 min	Increased total phenolic content; Enhanced DPPH radical scavenging activity from 59% to 67%; Improved the oxidative stability of the water-based nanoemulsion.	[114]
*Passiflora caerulea* L. seeds	Phenolics	NADES ^3^ ChCl: Fru: H_2_O	40 kHz, 200 W (RP), 60 min, 45 °C	Improved the extraction rate and types of phenolic compounds; Increased bioaccessibility of gallic acid; Increased antimicrobial activity.	[115]

^1^ Reported power with the basis not specified in the original study. ^2^ Power density. ^3^ Natural deep eutectic solvent.

**Table 4:** Current evidence and future directions for power ultrasound in emerging food biomanufacturing platforms.

Emerging Platform	Current Applications	Future Directions	References
Precision fermentation and microbial cell factories	Low-intensity ultrasound increased ε-polylysine production in a *Streptomyces albulus* strain, alongside changes in intracellular ATP and biosynthetic enzyme activities.	Evaluate feedstock pretreatment to improve nutrient accessibility before inoculation. Assess low-intensity sonication during cultivation under oxygen or nutrient transport limitations, while monitoring genetic stability, cell viability, and target-product accumulation. Evaluate post-harvest sonication for intracellular product recovery.	[143,144,145]
Biomass fermentation for microbial protein production	The cited ultrasound studies focus on downstream recovery and modification of microbial biomass components. Ultrasound released yeast proteins and fungal polysaccharide–protein complexes. Ultrasonication combined with pH shifting modified yeast-protein structure, functionality, in vitro digestibility, and bioactivity.	Determine cultivation-stage exposure conditions that support biomass production while maintaining cell viability. Optimize post-harvest disruption for protein recovery and in vitro digestibility. Tailor protein modification to the functionality required in food applications.	[146,147,148]
Synthetic microbial communities for directed food fermentation	The cited platform studies address community stability and flavor formation, but do not demonstrate ultrasound-assisted fermentation. Benefits observed in single-strain cultures cannot be assumed for designed multispecies communities.	Evaluate substrate pretreatment before inoculation to improve nutrient availability. Determine how inoculum or in-process sonication affects individual strains and community composition. Track strain abundance, metabolic interactions, community recovery, and target-product formation.	[149,150]
Cell-free and multi-enzyme biomanufacturing	Reported ultrasound applications include lysate preparation for cell-free expression and enzyme immobilization. Sonication energy during *Escherichia coli* lysis affected subsequent protein-expression capacity. Ultrasound enhanced enzyme immobilization using a tyrosine-tag biocrosslinking strategy.	Optimize lysate preparation for the intended expression system while preserving its protein-synthesis capacity. Evaluate ultrasound-assisted enzyme immobilization and transport enhancement in immobilized or multiphase cascades. Measure target-protein yield in cell-free expression, and productivity, selectivity, cofactor stability, and operational stability in enzyme cascades.	[151,152,153]

**Table 5:** Positive, neutral, and negative effects of power ultrasound in fermentation, aging, enzymatic hydrolysis, and extraction.

Process	Ultrasound Effects	Number of Publications	Main Findings	References
Fermentation	Positive	16	Increased microbial biomass by 14.92–127.03%.	[40,43,46,158]
			Increased target product contents (ethanol by 30.79%, ε-polylysine by 28.45%, and peptides by 15.05–61.61%).	[1,47,145]
			Shortened fermentation time or accelerated acidification.	[26,33,41]
			Increased phenolic release and enhanced in vitro antioxidant activity (total phenolics increased by 9.83–19.05% and total flavonoids by 4.92–54.17%).	[28,29,34]
			Improved water-holding capacity, structure, and texture (yogurt water-holding capacity increased by 10.00–11.51%, while syneresis decreased by 23.33–26.67%).	[33,35,53]
	Neutral	2	No significant effects on microbial biomass or the sensory quality of fermented products.	[159,160]
	Negative	4	Reduced the antioxidant or antimicrobial activity of fermented products.	[28,161]
			Reduced microbial biomass.	[162,163]
Enzymatic hydrolysis	Positive	16	Increased enzyme catalytic activity by 5.8–93%.	[78,80,92,93]
			Improved saccharification or sugar recovery (fermentable sugars from brewer’s spent grain increased by 113–127%; reducing sugars in another system by 22%; saccharification after combined pretreatment increased by 4.63 percentage points).	[74,86,100]
			Increased protein hydrolysis and peptide or protein recovery (degree of hydrolysis increased by 9.67 percentage points, peptides by 8.25–42.43%, and protein recovery by 6.08%).	[2,83,96]
			Enhanced in vitro antioxidant and enzyme-inhibitory activities of hydrolysates (ACE inhibition increased by 63.30%, lipoxygenase inhibition by more than 100%, and α-amylase inhibition by 43%).	[2,76,89]
			Improved functional or digestive properties of hydrolysis products (solubility, foaming capacity, and emulsifying activity increased by 19.89%, 33.33%, and 38.74%, respectively; resistant starch content increased by 19.90%).	[83,85,99]
	Neutral	4	No significant effects on the optimum temperature or pH of enzymes.	[94,164]
			No significant effects on free amino acid content, soluble protein content, or degree of hydrolysis.	[89,165]
	Negative	5	Reduced enzyme activity.	[14,78,166]
			Reduced the water-holding capacity of hydrolysates.	[98,167]
Aging	Positive	6	Increased ester concentrations by 7.3–286.78% and promoted the formation of other aroma compounds.	[13,64,67,68]
			Improved selected color parameters and promoted pigment formation through polymerization.	[60,64]
			Increased selected phenolic contents and in vitro antioxidant capacity.	[64]
			Accelerated the development of aging characteristics.	[68,69]
	Neutral	1	No significant changes in total esters, higher alcohols, terpenes, or tannins.	[168]
	Negative	3	Reduced total pigment and anthocyanin contents.	[61,169]
			Increased astringency.	[63]
Extraction	Positive	11	Increased extraction yields or recovery of target compounds by 8.65–52.94%.	[15,108,109,120]
			Shortened extraction time and accelerated compound release and diffusion.	[109,116,170]
			Enhanced in vitro antioxidant activity of extracts (DPPH scavenging increased by 8 percentage points; DPPH IC50 of polysaccharides obtained by ultrasound-assisted enzymatic extraction decreased by 27.08–29.40%).	[111,112,171,172]
			Improved selected functional properties of extracted proteins, including solubility, emulsifying activity, foaming capacity, and oil-holding capacity (sunflower protein oil absorption increased by 21.07%).	[116,148]
	Neutral	4	No significant effects on the extraction yields of target compounds, including lipids, curcumin, phenolics, and licorice constituents.	[173,174,175,176]
	Negative	8	Promoted degradation of target compounds, including curcumin, anthocyanins, and flavonoids.	[177,178,179]
			Reduced the immunological or antioxidant activity of extracts.	[128,180,181]
			Promoted lipid oxidation.	[17,110]

## Data Availability

No new data were created or analyzed in this study. Data sharing is not applicable to this article.
